# Exploring the anticancer and antioxidant properties of *Vicia faba* L. pods extracts, a promising source of nutraceuticals

**DOI:** 10.7717/peerj.13683

**Published:** 2022-08-17

**Authors:** Jessica Ceramella, Chiara La Torre, Michele De Luca, Domenico Iacopetta, Alessia Fazio, Alessia Catalano, Gaetano Ragno, Pasquale Longo, Maria Stefania Sinicropi, Camillo Rosano

**Affiliations:** 1 Department of Pharmacy, Health and Nutritional Sciences, University of Calabria, Arcavacata di Rende, Cosenza, Italy; 2 Department of Pharmacy-Drug Sciences, University of Bari “Aldo Moro”, Bari, Italy; 3 Department of Chemistry and Biology, University of Salerno, Fisciano, Italy; 4 Proteomics and Mass Spectrometry, IRCCS Policlinico San Martino, Genova, Italy

**Keywords:** Nutraceutics, Anticancer, Antioxidant, Phytochemicals, Tubulin, Docking simulations, Sk-Mel28 Melanoma cell lines, *Vicia faba* L.

## Abstract

**Background:**

Pulse crops are considered the major sources of proteins, dietary fiber, micronutrients, and bioactive phytochemicals. Among the numerous pulse crops, broad beans (*Vicia faba* L.) have received particular attention due to their nutraceutical, functional and economic importance. Our attention was mainly focused on the broad bean pods (VFs), which are the primary by-product of the domestic and industrial processing of broad beans and an attractive source of valuable ingredients.

**Methods:**

In order to investigate the VFs properties, the flours from broad beans of three different harvest periods were extracted with acetone, methanol and 70% aqueous ethanol and the dried extracts were analyzed, qualitatively and quantitatively, and tested for their antioxidant through DPPH and ABTS assay and anticancer activities using the MTT assay and immunofluorescence analysis.

**Results:**

The VF extracts demonstrated a good *in vitro* radical scavenging activity from the first stage of collection of all the *V. faba* L. extracts. Additionally, the extracts were tested for their cytotoxicity against a panel of cancer and normal cells and the outcomes indicated the ethanol extract as the most active against the melanoma cell line Sk-Mel-28, without affecting the viability of the normal cells. Finally, we found out that the ethanol extract interfered with the microtubules organization, leading to the cancer cells death by apoptosis.

## Introduction

In many industrialized countries, the wastes and by-products from vegetables, usually generated during the harvesting or post-harvesting stages, are sometimes re-employed as animal feed sources. The inedible parts are discarded and represent an important issue for the environment. Therefore, the study of the chemical composition and biological activities of these waste materials is crucial for encouraging their adequate reuse in different medical applications. In particular, *Vicia faba* L. is a species of the Fabaceae family, commonly called fava bean, cultivated and consumed worldwide. The production of *Vicia faba* L. pods (broad bean pods, VFs), during their domestic and industrial processing, amounts to around 70% of the starting material, which disposal represents an important problem in terms of costs and waste of valuable nutrients (Valente et al., 2018). Indeed, several data reported that VFs are an attractive source of soluble sugars, fatty acids, minerals, proteins and, as well, dietary fiber (Fazio et al., 2020) and important non-nutrients as, for instance, flavonoids and polyphenols (Borowska, Giczewska & Zadernowski, 2003; Pritchard, Dryburgh & Wilson, 1973). The latter possess health promoting and disease preventive properties against different chronic-degenerative diseases, including cancer (González-Montoya, Cano-Sampedro & Mora-Escobedo, 2017). Not many literature studies reported the biological activity of *Vicia faba* L. bean pods. A recent one demonstrated that the methanol extract of VFs was found as the most active in term of antimicrobial and antioxidant activities and it could also be considered useful for the management of diabetes (Mejri et al., 2018). It was also reported that several flavonoids isolated from VFs reduced the viability of MCF-7 breast cancer cells, inducing mitochondrial-dependent apoptosis (El-Feky, Elbatanony & Mounier, 2018). We recently demonstrated that VFs could serve as a good source for the extraction of nutraceuticals, such as glucans and pectins (Fazio et al., 2020). In this article, we investigated the antioxidant and anticancer properties of VFs extracts at three different collection stages, obtained during the β-glucan isolation procedure, using acetone, methanol and ethanol 70%. All the three extracts were studied in order to evaluate their qualitative and quantitative content in flavonoids and polyphenols. These studies suggested that the highest amount of polyphenols resides in 70% ethanolic and methanolic extracts from the first stage of collection (VFIe and VFIm, respectively), which were also the most efficient in inhibiting DPPH and ABTS radicals by 50%. Although there are not many data on VFs anticancer activity, we exposed a broad panel of cancer cells (breast, melanoma, uterine and colon cancer cells) to all the extracts. We individuated two extracts (VFIe and VFIm) with the highest anticancer activity, mostly against the Sk-Mel-28 melanoma cells, without affecting the growth of the normal cell lines used in our assay. The exposure of Sk-Mel-28 to both the extracts produced the cancer cells death by apoptosis. In addition, the dramatic cell morphology change noticed after the exposure to VFIe and VFIm extracts, pushed us to study *in vitro* their involvement in regulating the cell cytoskeleton components, particularly the tubulin. Indeed, we proved that both the extracts were able to interfere with tubulin organization, in a similar fashion to vinblastine, a well-known anti-tubulin agent and a clinically used drug, chosen as reference molecule for its similar mechanism of action to that of the studied extracts. *In. silico* studies confirmed that all the phenolic compounds identified in *Vicia faba* L. extracts by HPLC were able to bind, with good binding energies, the three-dimensional structure of tubulin. These findings are seminal for a better and desirable comprehension of the additional re-use and valorization of agro-food wastes, a valuable and worthy source of preventive/curative compounds and nutraceuticals.

## Materials and Methods

### Chemicals and reagents

Analytical grade acetone, methanol, ethanol and water (VWR International s.r.l., Milan, Italy) were used for the extraction of phenolic and flavonoid compounds. Trolox (6-hydroxy-2,5,7,8-tetramethilchroman-2-carboxylic acid, 97%), DPPH (2,2-Diphenyl-1-picrylhydrazyl, 95%), ABTS (2,2′-azino-bis-(3-ethylbenzothiazoline-6-sulphonic acid) diammonium salt, potassium persulphate, potassium acetate, aluminium chloride and Folin-Ciocâlteu reagent were obtained from Sigma-Aldrich (Milan, Italy).

Analytical standard grade vanillic acid (97%), ellagic acid (97%), *p*-coumaric acid (98%), ferulic acid (99%), quercetin (98%), gallic acid (98%) were also purchased from Sigma-Aldrich (Milan, Italy). HPLC gradient grade methanol and water (VWR International s.r.l., Milan, Italy) was used as chromatographic mobile phase. Formic acid (99%; Sigma Aldrich, Milan, Italy) was used to acidify the aqueous mobile phase, prepared with high-purity water. All samples were filtered through a syringe filters in PVDF, pore size 0.45 µm (Millipore, SER.DIA s.r.l., Reggio Calabria, Italy).

### Plant material

The *Vicia faba* L. used were grown at a locality of Acri (39°29′38″04 Nord and 16°23′4″56 Est, Calabria, Southern Italy). The harvest was done in the period from April to May 2018 three times from the same soil at a time interval of 20 days from each other. The seeds were removed and the pods used were named according to the harvest period as VFI (first collection, 15–18 cm length, 4–5 small seeds inside), VFII (second collection, 20–25 cm length, 7–8 intermediate seeds inside), and VFIII (third collection, 25–30 cm length, 9–10 large seeds inside). The pods were cut into small pieces with a knife and freeze dried (Telstar freeze-dryer, mod. Cryodos), ground to a fine powder using a 60-mesh screen (particle size equal to 250 μm) and stored at −20 °C, before the extraction.

### Preparation of the extracts

The flour was first extracted with acetone, then with methanol and finally with 70% aqueous ethanol. These solvents were used in the preliminary stage of the β-glucan isolation procedure in order to remove soluble compounds from the flour (Fazio et al., 2020). In particular, the use of these specific solvents was required as per protocol to remove lipids and soluble materials such as vitamins, polyphenols and others and then to facilitate the complete separation of fiber from other compounds that may be present. Dry matter (3 g) was weighed in 50 mL capped plastic tubes and 30 mL (g 10 mL^−1^) of acetone were added. The suspension was left under stirring for 1 h at room temperature, then separated from the solid residue by centrifugation at 5,000 rpm for 15 min. The supernatant was filtered using sintered glass Buchner funnel (4 µm pore size) and collected in a previously weighed flask. The extraction was repeated three times on the resulting solid and filtrates combined. The solid residue from these extractions was extracted with methanol (3 × 30 mL) and centrifugated each time. The last extractions were performed on the solid residue using 70% aqueous ethanol (3 × 30 mL). The organic solvent from the three filtrates (acetone, methanol, and 70% aqueous ethanol) was separately removed under reduced pression. The crude extracts were conserved at −20 °C under N_2_. The weight of each extract was expressed as the average value ± standard deviation.

### Total phenolic content (Folin-Ciocâlteu assay)

The total phenolic content (TPC) was determined according to the modified Folin-Ciocâlteu method adapted by Plastina, Fazio & Gabriele (2012).

Briefly, 0.1 mL of the sample (1 mg mL^−1^DMSO) were mixed with 0.5 mL of 1:10 diluted Folin–Ciocâlteu reagent and 2 mL of distilled water. The mixture was shaken for 4 min and then 0.4 mL of a 10% sodium carbonate solution (Na_2_CO_3_), were added and the mixture was stirred in the dark at room temperature. After 30 min under magnetic stirring the absorbance was measured at 765 nm using UV-Vis spectrophotometer (Ultrospec 2100 Pro; Amersham Biosciences/GE Healthcare, Amersham, UK), using an appropriate blank for background subtraction. Gallic acid standard solutions at five different concentrations in the range 1–600 µg mL^−1^DMSO were prepared and used to construct a calibration curve (r^2^ = 1; y = 3.394x − 0.0179).

The TPC in each extract was the mean of three assays expressed as mg of gallic acid equivalents per gram dry extract (mg GAE g^−1^ DW).

### Total flavonoid content

The total flavonoid content (TFC) was determined using a colorimetric method (Fazio et al., 2018). For each extract, 0.3 mL of the sample (1 mg mL^−1^DMSO) were dissolved in 0.9 mL of MeOH. Then, 0.06 mL of 10% AlCl_3_ solution, 0.06 mL of 1 M solution of potassium acetate and 1.68 mL of distilled water were added. After 30 min of incubation in the dark, under constant magnetic stirring at room temperature, the absorbance was measured at 420 nm using a UV-Vis spectrophotometer (Ultrospec 2100 Pro; Amersham Biosciences/GE Healthcare, Amersham, UK) against a blank. Three analyses were carried out for each sample. Quercetin standard solutions within the 12.5–100 µg mL^−1^ DMSO range concentrations were prepared to construct a calibration curve (r^2^ = 0.9993; y = 0.0073x + 0.001). The TFC was expressed as µg of quercetin equivalents per gram of dry extract (µg QE g^−1^ DW).

### Radical scavenging activity

Antioxidant activity of the ethanolic, methanolic and aqueous extracts from leaves was determined by DPPH and ABTS assays.

#### Free radical scavenging activity (DPPH assay)

DPPH free radical scavenging capacity of the *Vicia faba* L. extract was determined according to the procedure employed in a previous study (Plastina, Gabriele & Fazio, 2018). Briefly, 0.1 mL of sample (1 mg mL^−1^, 0.5 mg mL^−1^, 0.1 g mL^−1^, 0.05 mg mL^−1^) was mixed with 0.1 mL of DPPH solution (1 mM) and 2.8 mL of MeOH. After an incubation time of 30 min, under magnetic stirring at room temperature, the absorbance was measured at 517 nm using a UV-Vis spectrophotometer (Ultrospec 2100 Pro; Amersham Biosciences/GE Healthcare, Amersham, UK). The experiments were carried out against a blank (3 mL of MeOH) and a control (2.9 mL of MeOH, 0.1 mL DPPH solution). All the determinations were performed in three different replicates.

The antioxidant activity was calculated as percentage of DPPH inhibition (%I_DPPH_) and determined according to the equation:

$\eqalign{\text{%}I_{DPPH} = [(Absorbance \; of \;the\; control\; - average \;absorbance \;of\; the\; sample)/ \cr\quad Absorbance \;of\; the\; control]\; \times \;100}$

Each %I_DPPH_ value corresponds to the average of the three results ± standard deviation.The radical scavenging activity was also expressed as Trolox equivalent antioxidant capacity (TEAC) and a standard curve was created using 0.001–1 mg mL^−1^ Trolox solutions (r^2^ = 0.9878; y = −0.0014x + 0.3444). The EC_50_ values of all the extracts against DPPH were evaluated by GraphPad Prism 8 Software.

#### Free radical scavenging activity (ABTS assay)

ABTS was well soluble in both aqueous and organic solvents, so this method can be used extensively for the determination of antioxidant activity for both hydrophilic and lipophilic compounds (Leporini et al., 2019). This assay is based on the scavenging of 2, 2′-azino-bis (3-ethylbenzothiazoline-6-sulphonate) radical cation (ABTS·^+^).

ABTS·^+^ radical cation was prepared by mixing a 7 mM ABTS solution and 2.45 mM potassium persulfate, and allowing the mixture to stand in darkness at room temperature for about 12–16 h. The radical cation generated was stable for the next 48 h and is characterized by an intense green/blue color. The ABTS solution has been diluted adequately so that its absorbance reached the value of 0.700–0.730 at 734 nm.

For each extract four solution of different concentrations (1.0, 0.5, 0.1, 0.05 mg mL^−1^ DMSO) were prepared. A total of 3 mL of ABTS radical solution and 0.030 mL of each sample were mixed and after 5 min of incubation at room temperature in the dark the absorbance was recorded at 734 nm, against a control (3 mL of EtOH 70%, 0.030 mL of DMSO). The results were expressed as a percentage of radical inhibition (% I_ABTS_), according to the formula:

$\eqalign{\text{%}I_{ABTS} = [(Absorbance\; of\; the\; control\; -average\; absorbance \;of\; the\; sample)/ \cr\quad Absorbance \;of\; the\; control] \times \mathit{100}.}$

The value was the average of the three results ± standard deviation. The results were also expressed as µg of Trolox equivalent (TAEC), calculated by constructing a calibration curve (r^2^ = 0.9982; y = 0.0933x + 0.704). The EC_50_ values of all the extracts against ABTS·^+^ were evaluated by GraphPad Prism 8 Software.

### Qualitative and quantitative analysis of the phenolic and flavonoid compounds by HPLC-DAD

The quantitative analysis of the extracts was performed by high performance liquid chromatography (Shimadzu, Kyoto, Japan) equipped with Auto Sampler (SIL 20A), pumps (LC 20AD), oven (CTO-10AS vp), diode array-detector (SPD M20A) and a system controller CMB-20A. Mediterranea SEA (Terrassa, Spain) C-18 column (4.6 mm × 25 cm, 5 µm) was used for the analysis. The extracts were filtered through membrane filter (0.45 µm) into HPLC amber vials. The mobile phase was a mixture of water containing 1% of formic acid (A) and MeOH (B). All the analyses were performed in gradient elution mode as follows: 0 min, 10% B; 0–10 min, 20% B; 10–20 min, 30% B; 20–30 min, 40% B, 30–40 min, 50% B, 40–50 min, 60% B, 50–60 min, 70% B, 60–70 min, 80%, 70–80 min, 90% B, 80–90 min, 100% B, 90–120 min, 100% B, 120–125 min, 10% B. The flow rate and column temperature were 0.6 mL min^−1^ and at 29 °C, respectively.

The qualitative analysis was performed compounds by comparing the chromatograms of the extracts with the reference compound ones. In addition, in order to assign the elution time of each compound, analyses were conducted on each extract that was enriched with the standards one by one. The individual polyphenolic compounds were quantified by the external standard method using the calibration curves of the following commercial standards: vanillic acid, ellagic acid, quercetin, *p*-coumaric acid, ferulic acid, chlorogenic acid and gallic acid which were detected at appropriate wavelengths (280, 254, 365, 271, 325, 327 and 276 nm, respectively).

### Spectroscopic profiling *via* FTIR-ATR analysis

The infrared fingerprints of *Vicia faba* L. extracts were collected by using the Spectrum Two Fourier transform infrared (FTIR) spectrometer (Perkin Elmer Italia Spa, Milan, Italy), coupled with an attenuated total reflection (ATR) accessory consisting of a flat top-plate fitted with a ZnSe crystal. The ATR detector system was wiped before each analysis by using dry paper and methanol. The lab room air FTIR-ATR spectrum was used as background to verify the cleanliness, instrumental conditions, and interferences due to H_2_O and CO_2_. FTIR spectra of the samples, laid on the ATR top, were recorded in triplicate in the range 4,000–450 cm^−1^. Scan numbers and resolution were optimized at 32 scans and 4 cm^−1^, respectively (De Luca et al., 2016). A first selection of the range was necessary before data handling, variables matrix was cut keeping out the spectral region between 4,000 and 1,800 cm^−1^. The range 4,000–2,500 cm^−1^ contained mainly broad signals associated with the stretching vibrations of hydroxyl functions and the stretching vibrations of aromatic C-H that were poor in information about phenolic compounds. The range 2,500–1,800 cm^−1^ did not contain spectral relevant information too, apart from the band corresponding to CO_2_. Therefore, the remaining range 1,800–450 cm−^1^ was arranged in the matrix and subdued to multivariate data modelling (Abbas et al., 2017).

### Multivariate data analysis

Principal component analysis (PCA) allows to get the information available in the spectral fingerprint by projecting the samples and spectral variables on a set of new orthogonal variables called principal components (PCs). PCA was performed in order to estimate the metabolic differences in terms of phenolic compounds from the different fava bean samples and extracts (Marrelli et al., 2020). Original data consisting of the Fava bean extracts were arranged in a matrix where 27 samples were described by their FTIR-ATR spectra. PCA analysis was performed by using the SVD (Singular Value Decomposition) algorithm and the full cross-validation procedure used in order to select the optimal number of PCs. The Unscrambler X software version 10.5 from CAMO (Computer Aided Modelling, Trondheim, Norway) was used for the chemometric treatment of the spectral data (Spatari et al., 2017).

### Cell culture

All the cell lines used in this work were purchased from American Type Culture Collection (ATCC, Manassas, VA, USA). They were maintained at 37 °C in a humidified atmosphere of 95% air and 5% CO_2_ and periodically screened for contamination as already reported (Barbarossa et al., 2021; Ceramella et al., 2019; Iacopetta et al., 2020a).

### MTT assay

The *in vitro* antitumor activities of all the target compounds were determined by using the MTT (Sigma) assay, as already described (Sinicropi et al., 2018). Cells were exposed to the different extracts dissolved in DMSO at different concentrations (50, 100, 200, 400 and 500 µg/mL) for 72 h. Then MTT was added as already reported (Ceramella et al., 2021). The IC_50_ values were obtained by using GraphPad Prism 9 (GraphPad Software, La Jolla, CA, USA). Data are representative of three independent experiments performed in triplicate; standard deviations (SD) have been shown.

### TUNEL assay

Apoptosis was detected by the TUNEL assay, according to the guidelines of the manufacturer (CF^TM^488A TUNEL Assay Apoptosis Detection Kit; Biotium, Hayward, CA, USA), with some modifications. The cells were grown on glass coverslips and, after treatment with the most active extracts at their IC_50_ values, they were washed and methanol-fixed. The TUNEL reaction mixture containing the terminal deoxynucleotidyl transferase (TdT) were added, as already reported by Iacopetta et al. (2017). Samples were washed, incubated with DAPI (Sigma, 0.2 mg/mL) and then observed and imaged under a fluorescence microscope (Leica DM6000; 20× magnification, λ_ex/em_ maxima of 490/515 nm for CF^TM^488A or 350/460 nm for DAPI). Images are representative of three independent experiments.

### Immunofluorescence analysis

Cells were seeded in 48-well culture plates containing glass slides and then serum-deprived for 24 h and incubated with the most active extracts for 24 h (concentration equal to their IC_50_ value). The cells were incubated with primary antibody, as previously described (Ceramella et al., 2019; Sinicropi et al., 2021). The rabbit anti β-Tubulin were purchased from Santa Cruz Biotechnology and diluted 1:100 before the use. The secondary antibody used was Alexa Fluor® 546 conjugate goat-anti-mouse (1:500; Thermo Fisher Scientific, MA, USA). Leica DM 6000, 40× magnification was used in order to detect fluorescence and the images were processed using LAS-X software. All the experiments were performed in triplicate.

### Docking studies

The crystal structure Vinblastine in complex with a tetrameric assembly of Tubulin (Waight et al., 2016) (PDB code 5j2t), has been used as a protein target for our simulations. The atomic coordinates of the ligands we tested *in silico*, have been built and energy minimized using the program MarvinSketch (ChemAxon ltd, Budapest, Hungary). To better understand the binding modes to the protein target, the mechanisms of action of our molecules and to determine their binding energies, we used the program Autodock v.4.2.2. (Morris et al., 2009) and its graphical interface ADT (Sanner et al., 1999). We built a cubic searching grid composed by 126 nodes in each dimension, and we centred it on the protein centre. Nodes are spaced 0.375 Å each other. We run our simulations adopting the program default values. The protein target and the ligands were prepared using the ADT graphical interface: polar hydrogens were added, Kollman charged assigned to the ligands and eventually solvatation parameters added. Tubulin has been considered as a rigid object while all our ligands were taken as fully flexible. Our simulation was carried out as already reported (Iacopetta et al., 2020b; Lappano et al., 2015; Rosano et al., 2012; Viale et al., 2011).

### Statistical analysis

The experimental data regarding were expressed as means of three replications ± standard deviation. Statistical analyses were performed using GraphPad Prism 8.0.2 software and evaluated by two-way ANOVA followed by a multi-comparison Turkey test, and by multi-comparison Dunnett’s test to evaluate the significance of EC_50_ values against DPPH and ABTS. Significance was established at *p* values (*) < 0.05, (**) < 0.01, (***) < 0.001, and (****) < 0.0001.

## Results

### Recovery of the extracts from dry powder

**Figure 1:**
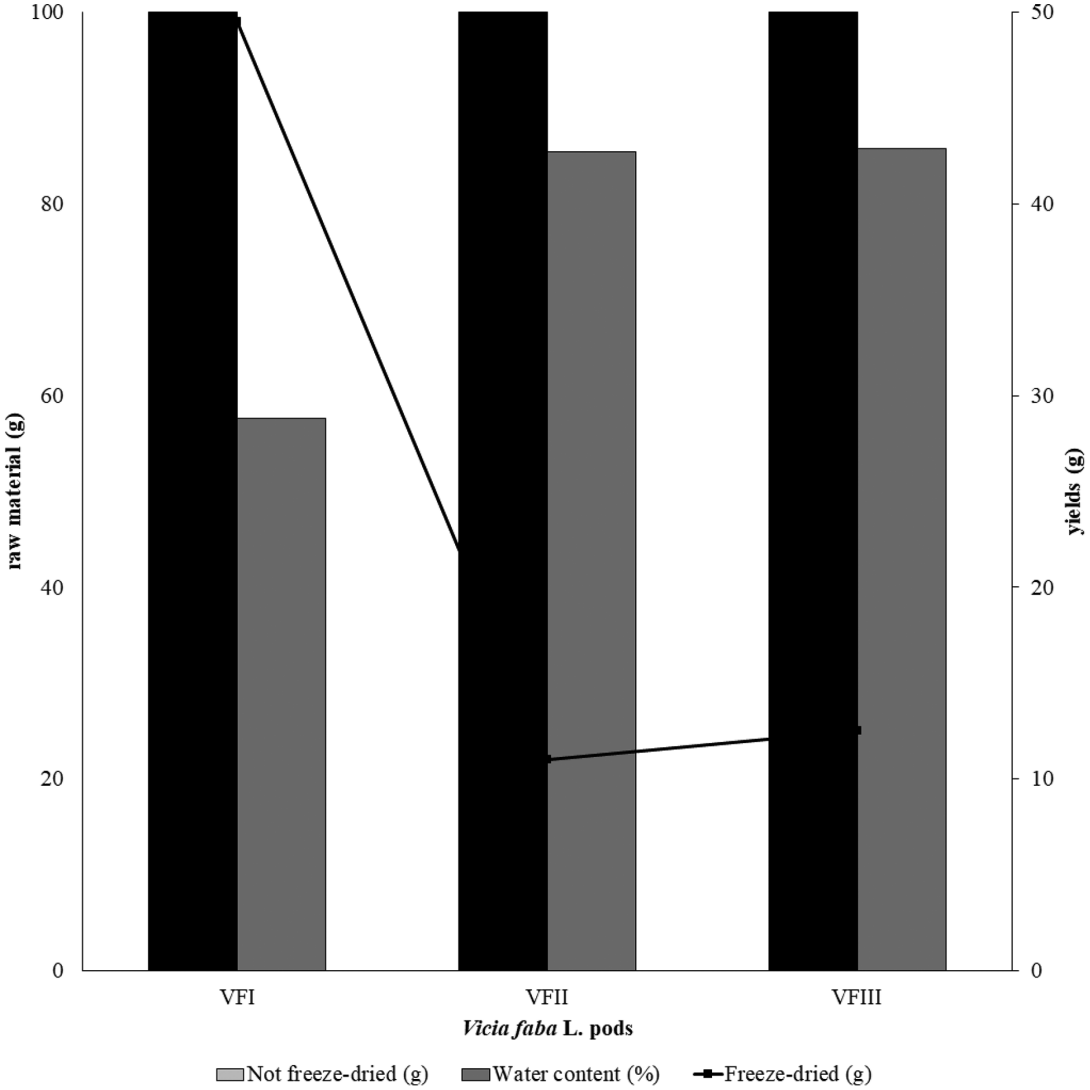
Recovery of the extracts from dry powder. Quantitative recovery (g) and water content from VFI, VFII and VFIII (100 g each without replicates).

**Figure 2:**
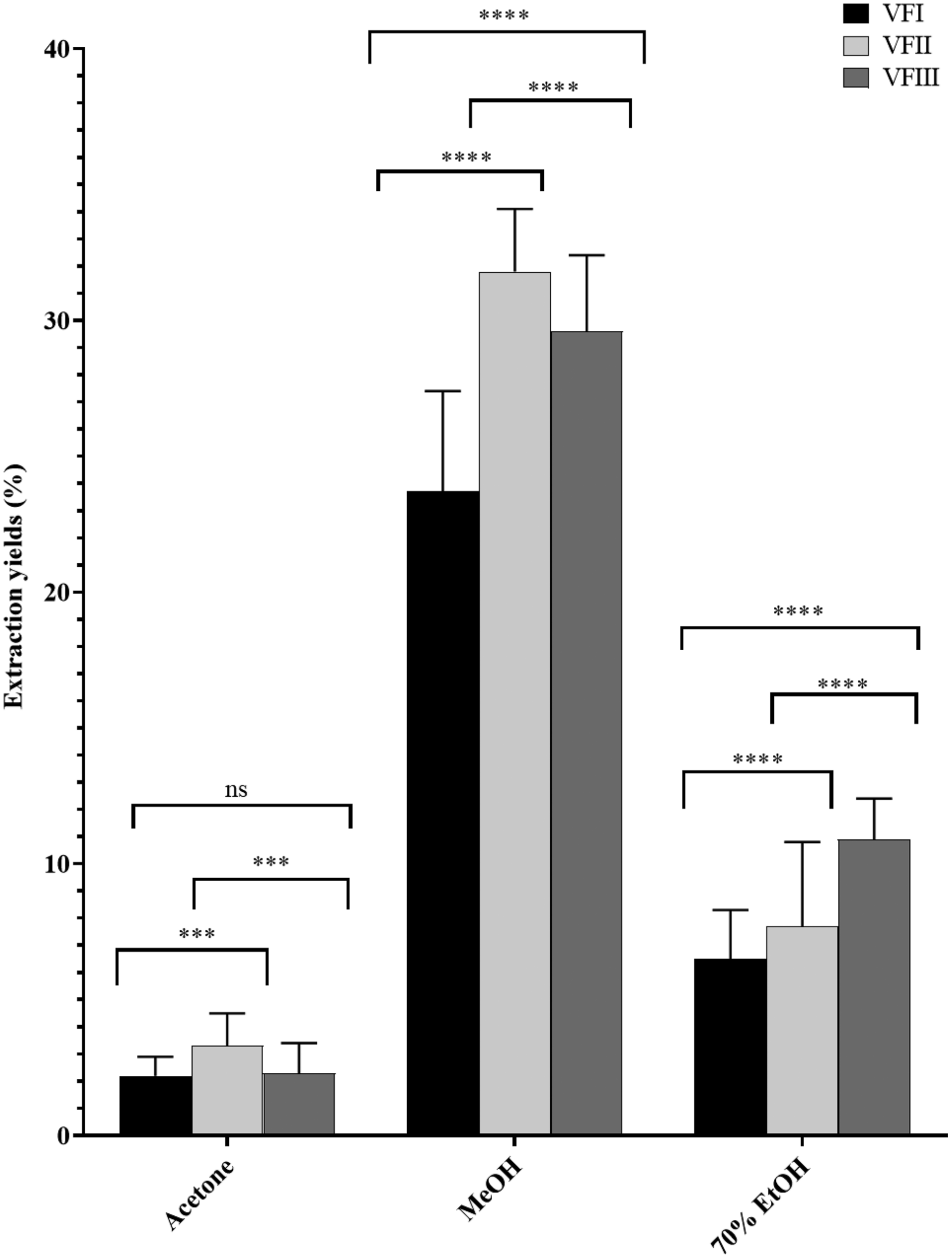
Extraction yields. Extraction yield (%) of acetone, methanolic and 70% ethanolic extracts. Histograms and error bars represent the mean of independent experiments performed in triplicate and standard deviation, respectively. Asterisks on histograms indicate that the mean values were statistically different (****p < 0.0001 and ***p < 0.001). Percentage yield of all the three extracts (acetone, MeOH and 70% EtOH) from each collection (VFI VFII and VFIII) were significantly different (****p < 0.0001).

The water content (%) and the yield of dry matter (g 100 g^−1^) were shown in Fig. 1. The yield (%) of each extract for all collections was reported in Fig. 2.

Data reported in Fig. 2 showed that the acetone extracts have a low yield in all three stages of collection (2.2–3.3%), but with greater recovery from VFII (VFII *vs*. VFII, VFIII *** *p* < 0.001). The major quantitative recovery occurs for methanol extracts (23.7–31.8%), with significance ****p < 0.0001 compared to acetone and ethanolic extracts (VFa, VFe) from each collection and also to methanolic extracts from the three stages of collection (VFIm, VFIIm, VFIIIm), which means that methanol has higher extraction efficiency despite its dielectric constant, being lower than that of 70% EtOH. The latter has an intermediate extraction efficiency compared to the other two solvents (6.5–10.9%), but significantly different (****p < 0.0001) in the ethanolic extract from the three harvests. Likely, as suggested from the Folin-Ciocâlteu assay, it has affinity for compounds that are not necessarily phenolic.

The quantitative recoveries from acetone and methanol extracts are more abundant in the medium stage of maturation (33 mg and 318 mg respectively) than from the ethanolic aqueous extract, for which the highest recovery is observed in the third collection (108.8 mg).

### Total phenolic content (TPC) by Folin-Ciocâlteu assay

**Figure 3:**
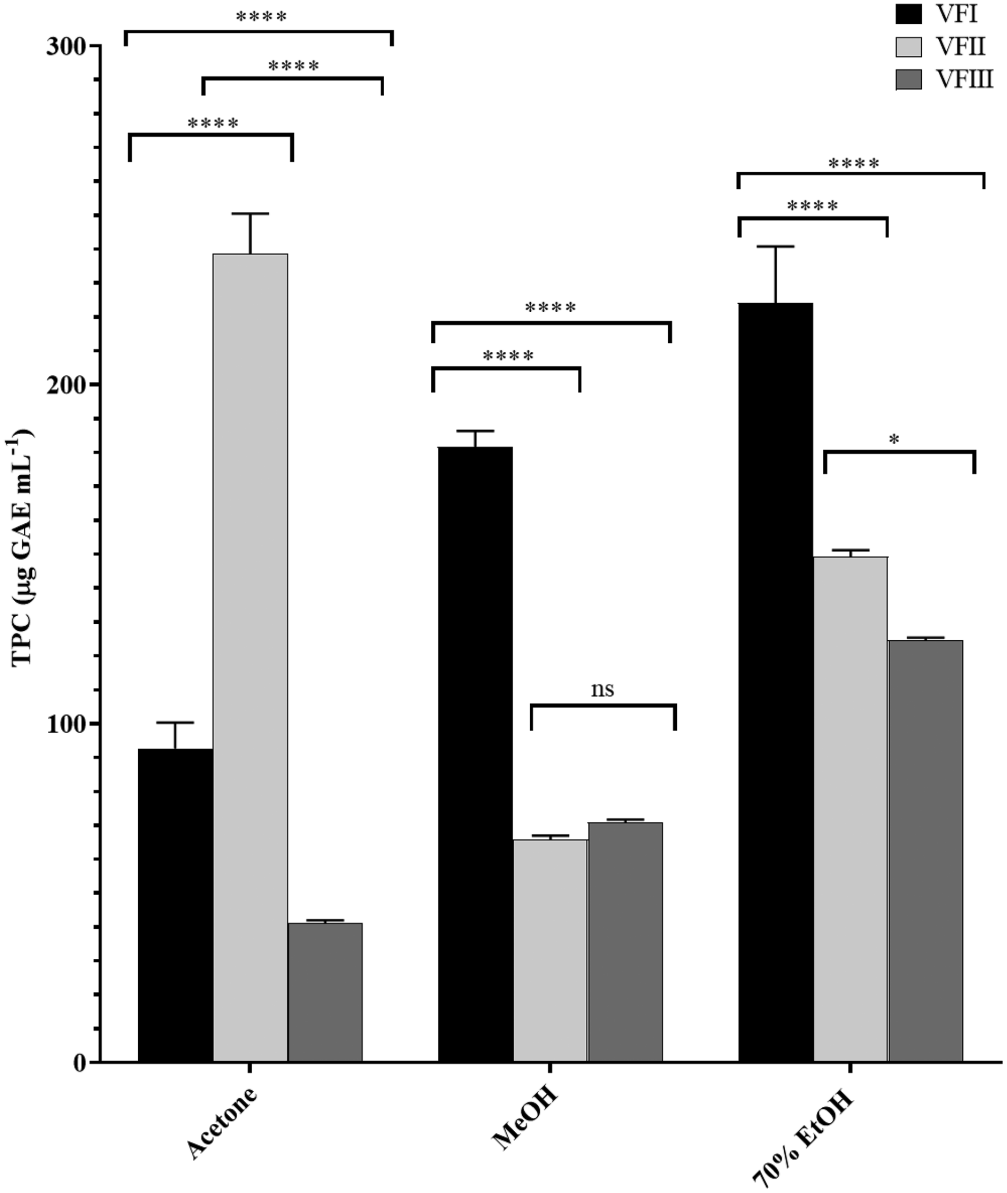
*Folin-Ciocâlteu assay*. TPC, expressed as µg GAE mL^−1^, of the three solvents (acetone, MeOH and 70% EtOH) from the three collections (VFI, VFII and VFIII). Histograms and error bars represent the mean of independent experiments performed in triplicate and standard deviation, respectively. Asterisks on the histograms indicate that the mean values were statistically different (****p < 0.0001 and *p < 0.05). The phenolic content of all the three extracts (acetone, MeOH and 70% EtOH) from each collection (VFI VFII and VFIII) were significantly different (****p < 0.0001).

The total phenolic content (TPC) in each extract was reported in Fig. 3 and was a function of the extraction solvent and collection time, decreasing with the maturation in ethanolic extract from 224.2 ± 16.6 (first collection) to 124.7 µg ± 0.7 GAE g^−1^_extract_ (third collection, VFI *vs*.VFII and VFIII ****p < 0.0001, VFII vs VFIII *p < 0.05) while increasing in acetone extract from 92.7 ± 7.6 µg GAE g^−1^_extract_ in the first collection to 238.6 ± 11.9 µg GAE g^−1^_extract_ in the second collection (****p < 0.0001). In the third collection it was halved compared to the content of stage I (****p < 0.0001).

In methanol, the initial content (181.7 ± 4.7 µg GAE g^−1^_extract_) was reduced by about one third the second collection (65.9 ± 1.1 µg GAE g^−1^_extract_, ****p < 0.0001) and, although without significance, it increased by 7% compared to the content of stage II (70.9 ± 0.8 µg GAE g^−1^_extract_).

The higher phenolic content was found in the acetone extract of the second collection (238.6 ± 11.9 µg GAE g^−1^_extract_), followed by the ethanolic extract of the first collection (224.2 ± 16.6 µg GAE g^−1^_extract_). The minor content of polyphenols was found in the acetone extract from VFIII (41.2 ± 0.8 µg GAE g^−1^_extract_).

The samples showed a progressive increase in the phenolic content with increasing polarity of the extraction solvent, apart from TPC in the second collection that is maximum in the acetone extract (238.6 ± 11.9 µg GAE g^−1^_extract_) despite the lower polarity of the solvent (ε = 20.7).

### Total flavonoid content (TFC)

**Figure 4:**
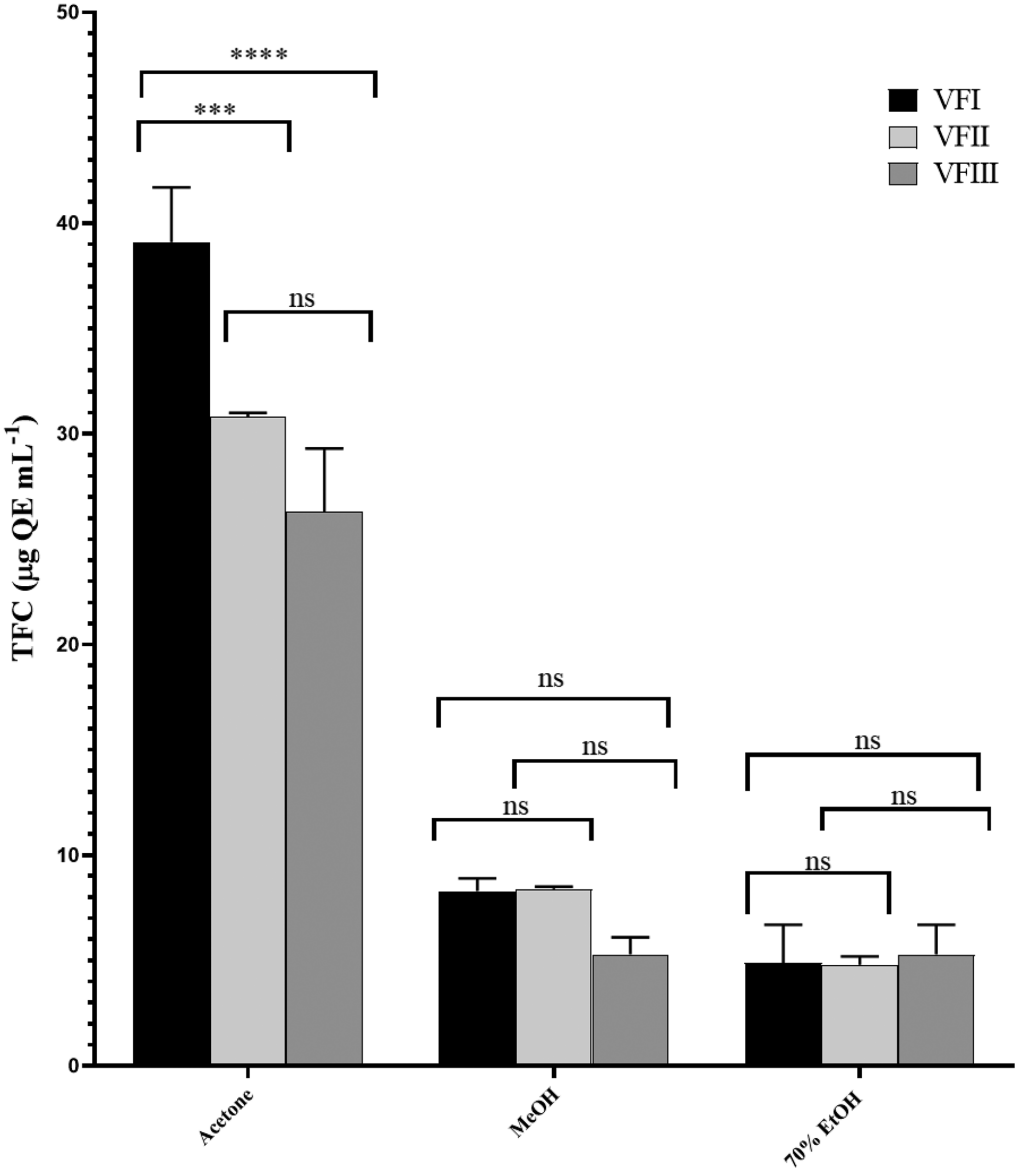
Total flavonoid content (TFC). TFC, expressed as µg QE mL^−1^, of the three solvents (acetone, MeOH and 70% EtOH) from the three collections (VFI, VFII and VFIII). Histograms and error bars represent the mean of independent experiments performed in triplicate and standard deviation, respectively. Asterisks on the histograms indicate that the mean values were statistically different (****p < 0.0001 and ***p < 0.001. TFC in the acetone extracts from all the three collections (VFI, VFII and VFIII) were significantly different (****p < 0.0001) compared the extracts in MeOH and 70% EtOH.

Figure 4 showed the TFC in all the extracts. Similarly to TPC, the TFC varied as a function of the extraction solvent and collection time. Flavonoid content in acetone extracts decreased as the ripening progresses (VFI *vs*. VFII ***p < 0.001, VFI *vs* VFIII ****p < 0.000, VFII *vs* VFIII *ns*), while TFC in ethanolic extracts increased from 4.9 ± 1.8 µg QE g^−1^_extract_ in the first collection to 5.3 ± 1.4 µg QE g^−1^_extract_ in the third collection, without any significance. In methanolic extracts, the initial content (8.3 ± 0.6 µg QE g^−1^_extract_) remained unchanged in the second collection (8.4 ± 0.1µg QE g^−1^_extract_) and then decreased in the last collection time (5.3 ± 0.8 µg QE g^−1^_extract_).

In all three harvesting, the TFC decreased with the increasing polarity and as the ripening progressed. The solvent with the best extraction efficacy against flavonoids was found to be acetone.

### Radical scavenging activity

Two different *in vitro* assays, DPPH and ABTS, were used to evaluate the changes in free-radical scavenging abilities of all extracts from VFI, VFII and VFIII.

#### DPPH assay

**Figure 5:**
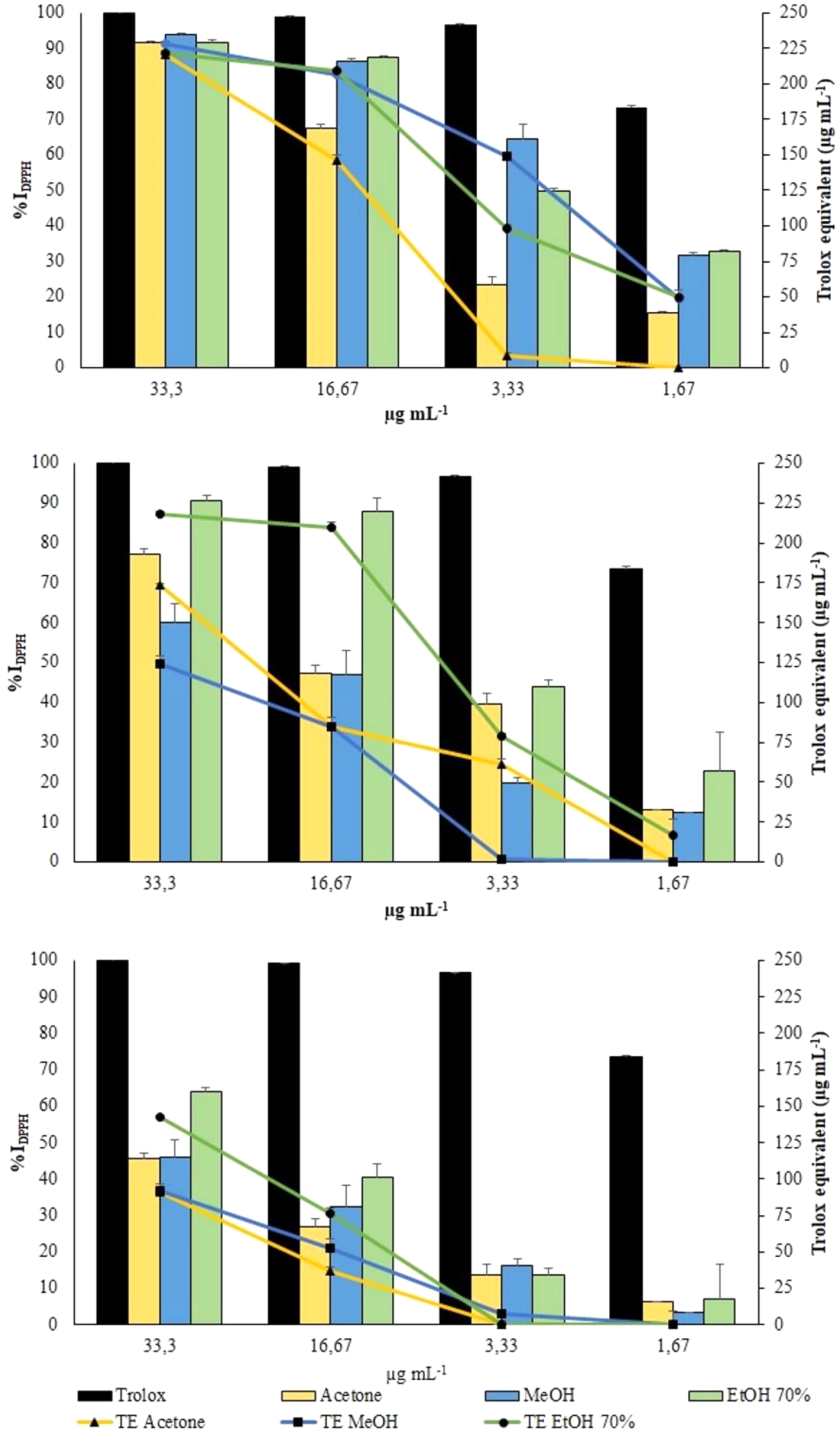
DPPH Assay. Changes in %I_DPPH_ (bars) and in TE (symbols) of all extracts from VFI, VFII and VFIII, respectively.

DPPH radical was scavenged by aqueous ethanol, methanol and acetone extracts from the three collections in a concentration-dependent manner (Fig. 5).

All the extracts from VFI exhibited the greatest antioxidant activity, exceeding 90% of DPPH inhibition (from 93.83% to 91.59%, that is 228.0 ± 2.0 µg TE g^−1^_extract_ to 220.8 ± 1.0 µg TE g^−1^_extract_) at the highest concentration tested (33.3 µg mL^−1^). The results obtained from the VFI sample can be correlated to the content of phenols and flavonoids. At the same concentration, only the ethanolic extract from VFII inhibited DPPH radical by 90.6 ± 0.4% (218.2 ± 1.2 µg TE g^−1^_extract_). The extracts from VFIII exhibited the lowest activity at all concentrations: at 33.3 µg mL^−1^, aqueous ethanolic, methanolic and acetone extracts inhibited DPPH radical by 63.8 ± 1.3% (142.7 ± 3.7 µg TE g^−1^_extract_), 46.2 ± 0.7% (124.6 ± 4.5 µg TE g^−1^_extract_) and 45.7 ± 0.7% (173.4 ± 1.5 µg TE g^−1^_extract_), respectively.

The EC_50_ values of aqueous ethanolic, methanolic and acetone extracts from VFI were found to be 3.1 ± 0.5, 2.5 ± 0.4 and 7.9 ± 0. 8 µg mL^−1^, respectively, indicating that the inhibition capacity of methanol against DPPH is comparable with the Trolox one, whereas that of acetone and ethanol is significantly lower than Trolox one (****p < 0.0001 and *p < 0.05, respectively)(Table 1). The EC_50_ values of all extracts from VFII and VFII are significantly higher than the Trolox one (****p < 0.0001 and ***p < 0.001 for VFIIe).

**Table 1:** EC_50_ values. EC_50_ values of the extracts against DPPH radical (expressed as µg mL^−1^).

Samples	VFI	VFII	VFIII
*Acetone*	7.9 ± 0.8****	10.9 ± 1.3****	39.6 ± 1.6****
*MeOH*	2.5 ± 0.4	18.2 ± 1.0****	35.1 ± 1.5****
*70% EtOH*	3.1 ± 0.5*	4.1 ± 0.6***	21.5 ± 1.2****

**Notes:**

The asterisks on the numbers indicate that means values were statistically different from the Trolox (EC_50_ = 0.84 ± 0.3).

**** *p* < 0.0001.

*** *p* < 0.001.

* *p* < 0.05.

#### ABTS assay

**Figure 6:**
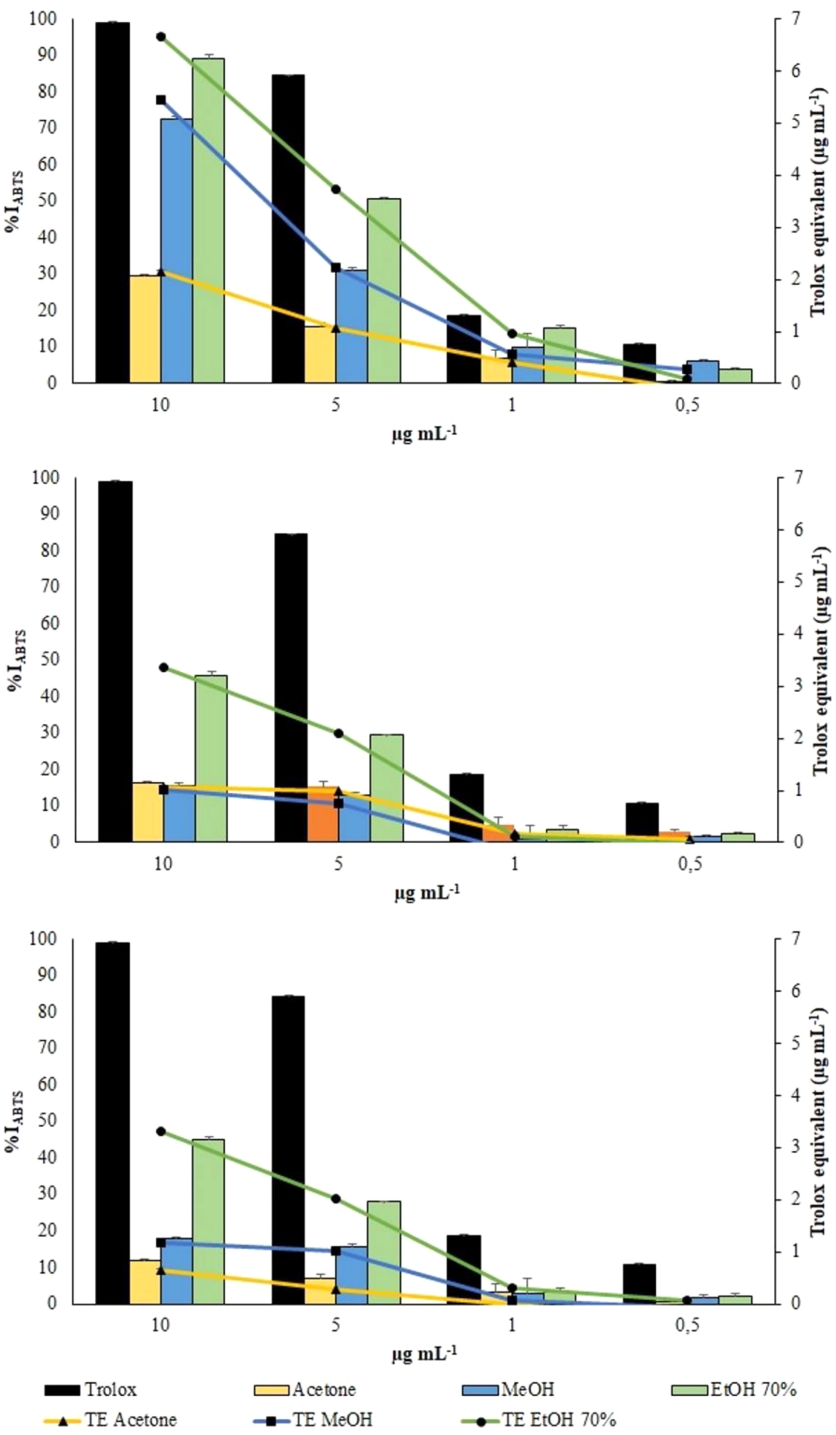
ABTS assay. Changes in %I_ABTS_ (bars) and in TE (symbols) of the extracts from VFI, VFII and VFIII, respectively

ABTS radical was scavenged by aqueous ethanol, methanol and acetone extracts from the three collections in a concentration-dependent manner (Fig. 6). However, all the extracts exhibited a lower scavenging activity toward the radical cation ABTS•^+^ than toward the radical DPPH.

Aqueous ethanolic and methanolic extracts from VFI inhibited the radical cation ABTS•^+^ by 89.3 ± 1.3 (6.7 ± 0.3 µg TE g^−1^_extract_) and 72.7 ± 0.2% (5.45 ± 0.1 µg TE g^−1^_extract_), respectively, at the highest concentration tested (10 µg mL^−1^). At the same concentration, acetone extracts from VFI showed the lowest percentage of radical inhibition (29.6 ± 0.6%, 2.1 ± 0.1 µg TE g^−1^_extract_). The EC_50_ values of aqueous ethanolic, methanolic and acetone extracts from VFI were found to be 3.8 ± 0.6, 6.8 ± 0.8, and 24.4 ± 1.4 µg mL^−1^, respectively, indicating that the inhibition capacity of 70% ethanol against ABTS•^+^ is comparable with the Trolox one, whereas that of acetone and ethanol is significantly lower than Trolox one (****p < 0.0001 and *** *p*< 0.001, respectively). The EC_50_ values of all extracts from VFII and VFII are significantly higher than the Trolox one (****p < 0.0001) (Table 2).

**Table 2:** EC_50_values of the extracts against ABTS radical. EC_50_values of the extracts against ABTS radical (expressed as µg mL^−1^).

Samples	VFI	VFII	VFIII
*Acetone*	24.4 ± 1.4****	40.7 ± 1.6****	70.8 ± 1.8****
*MeOH*	6.8 ± 0.8***	47.1 ± 1.7****	38.6 ± 1.6****
*70%EtOH*	3.8 ± 0.6	12.3 ± 1.1****	12.9 ± 1.1****

**Notes:**

The asterisks on the numbers indicate that means values were statistically different from the Trolox (EC_50_= 1.98 ± 0.3).

**** *p* < 0.0001.

*** *p* < 0.001.

### Qualitative and quantitative analysis of the phenolic and flavonoid compounds by HPLC-DAD

**Figure 7:**
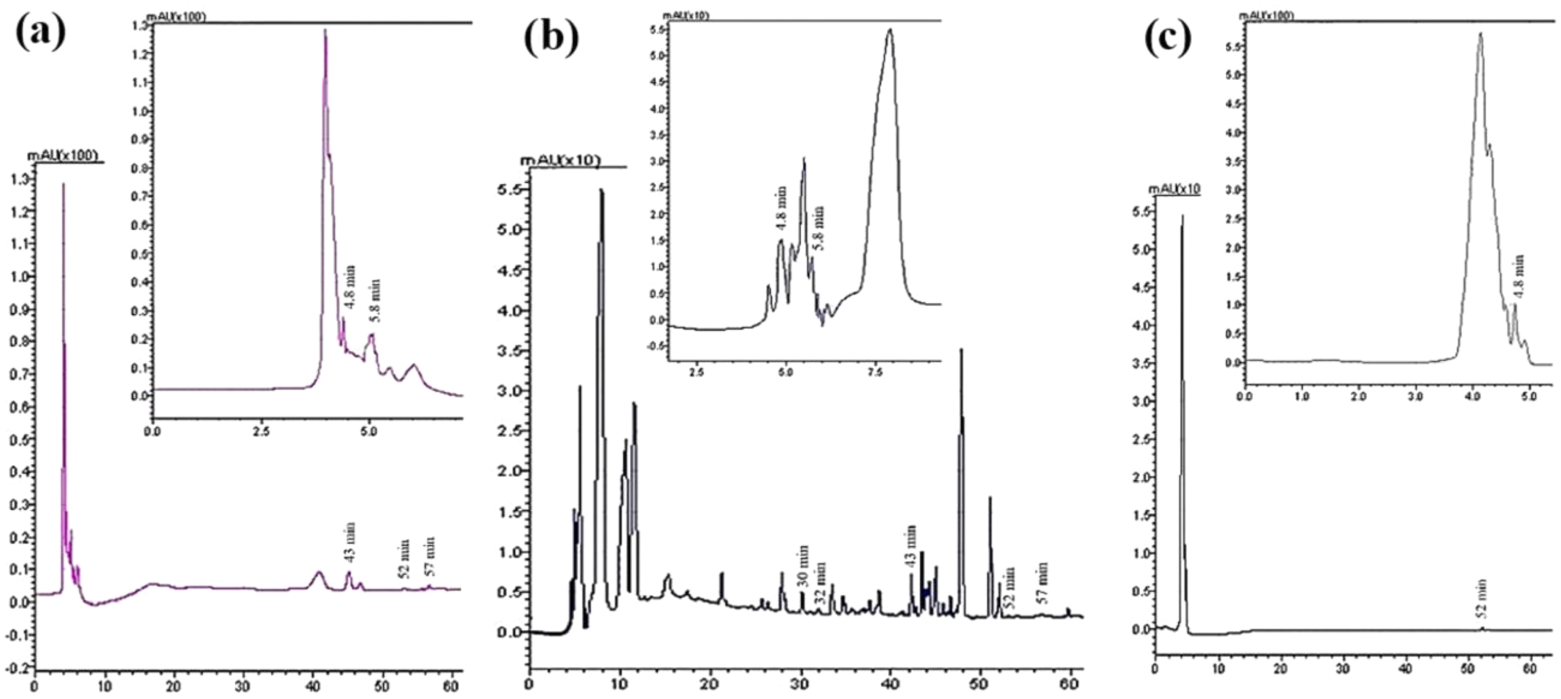
Qualitative and quantitative analysis of the phenolic and flavonoid compounds by HPLC-DAD. HPLC profile of (A) acetone, (B) methanolic and (C) 70% ethanolic extracts from VFII at λ = 280 nm. The identified compounds and corresponding retention time were: *p*-coumaric acid at 4.8 min, gallic acid at 5.8 min, chlorogenic acid at 30 min, vanillic acid at 33 min, ferulic acid at 43 min, ellagic acid at 52 min, and quercetin at 57 min.

**Figure 8:**
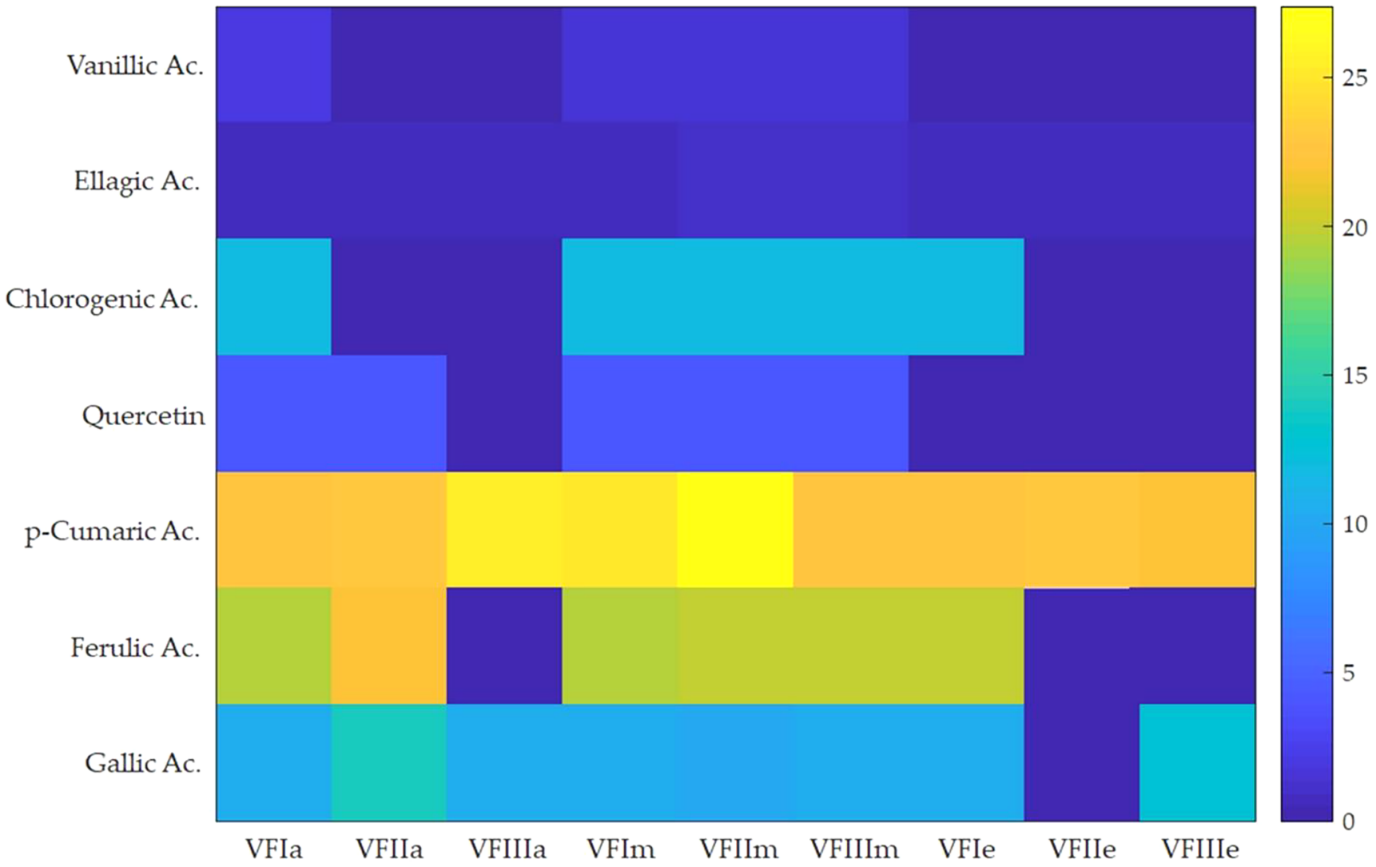
Heat-map of the contents of phenolic compounds. Heat map of HPLC quantification result of phenolic compounds in *Vicia faba* L. extracts. VFIa, VFIIa, VFIIIa were acetone extracts from VFI, VFII and VFIII, respectively. VFIm, VFIIm, VFIIIm were methanolic extracts from VFI, VFII and VFIII, respectively. VFIe, VFIIe, VFIIIe were aqueous ethanolic extracts from VFI, VFII and VFIII, respectively. As indicated in the color intensity scale of the figure legend, a yellow box indicates that the phenolic compound content was higher than the average level, and a blue box indicates that the phenolic compounds content was lower than the average level.

Seven compounds were identified and quantified by HPLC analyses in all samples (Fig. 7). The best quantitative and qualitative profile of polyphenols was recorded for VFI extracts, while the poorest extracts were from VFIII, except for the methanolic extracts from all three collections that contain the compounds identified in unchanged amount. In the methanolic and acetone extracts from VFI, seven compounds were identified among the standards, and five phenolics in the aqueous ethanolic extract (Table 3). The main compound in all the extracts was *p*-coumaric acid (21.9 ± 0.8–27.4 ± 5.1 µg g^−1^). Ellagic acid was contained in small amount, in all tested extracts (0.8 ± 0.1–1.3 ± 0.1 µg g^−1^). Chlorogenic acid was present only in methanolic extracts from all collections and in acetone extract from VFI. Quercetin and ferulic acid remained constant (about 4 and 19 µg g^−1^, respectively) in all the extracts except for the EtOH 70% extract from VFII and for acetone and EtOH 70% from VFIII, in which they were lacking. A heat-map was shown (Fig. 8) to visualize the contents of phenolic compounds and to highlight their distribution in the acetone, methanol and ethanolic aqueous extracts from the three different collections.

**Table 3:** List of compounds. Summary of the phenolic compounds and their content in *Vicia faba* L. extracts identified by HPLC.

	Extracts								
	**I (mg mL**^**−1**^ **± SD)**			**II (mg mL**^**−1**^ **± SD)**			**III (mg mL**^**−1**^ **± SD)**		
**Phenolic compounds**	*Acetone*	*MeOH*	*EtOH 70%*	*Acetone*	*MeOH*	*EtOH 70%*	*Acetone*	*MeOH*	*EtOH 70%*
*Vanillic acid*	2.11 ± 0.07	1.63 ± 1.44	–	–	1.63 ± 0	–	–	1.62 ± 0.02	–
*Ellagic acid*	0.81 ± 0.02	0.82 ± 0.01	0.77 ± 0.01	0.77 ± 0.02	1.27 ± 0	0.80 ± 0	0.80 ± 0.01	1.20 ± 0.02	0.82 ± 0.01
*Chlorogenic acid*	11.57 ± 0.02	11.58 ± 0.01	–	–	11.74 ± 0	–	–	11.75 ± 0.02	–
*Quercetin*	4.16 ± 0.01	4.14 ± 0	4.22 ± 0	4.22 ± 0	4.17 ± 0	–	–	4.13 ± 0.01	–
*p-coumaric acid*	22.36 ± 0.4	25.16 ± 1.16	22.90 ± 0.01	22.88 ± 0.02	27.40 ± 5.01	22.73 ± 0.03	25.60 ± 3.54	22.68 ± 0.03	21.91 ± 0.78
*Ferulic acid*	19.56 ± 0.01	19.63 ± 0.01	22.10 ± 0	22.10 ± 0	19.82 ± 0.40	–	–	19.91 ± 0.25	–
*Gallic acid*	10.39 ± 0	10.45 ± 0	13.85 ± 0	13.85 ± 0	10.21 ± 0.04	–	10.40 ± 0.35	10.41 ± 0.16	12.64 ± 0

### Principal Component Analysis (PCA) to infrared fingerprint characterization

**Figure 9:**
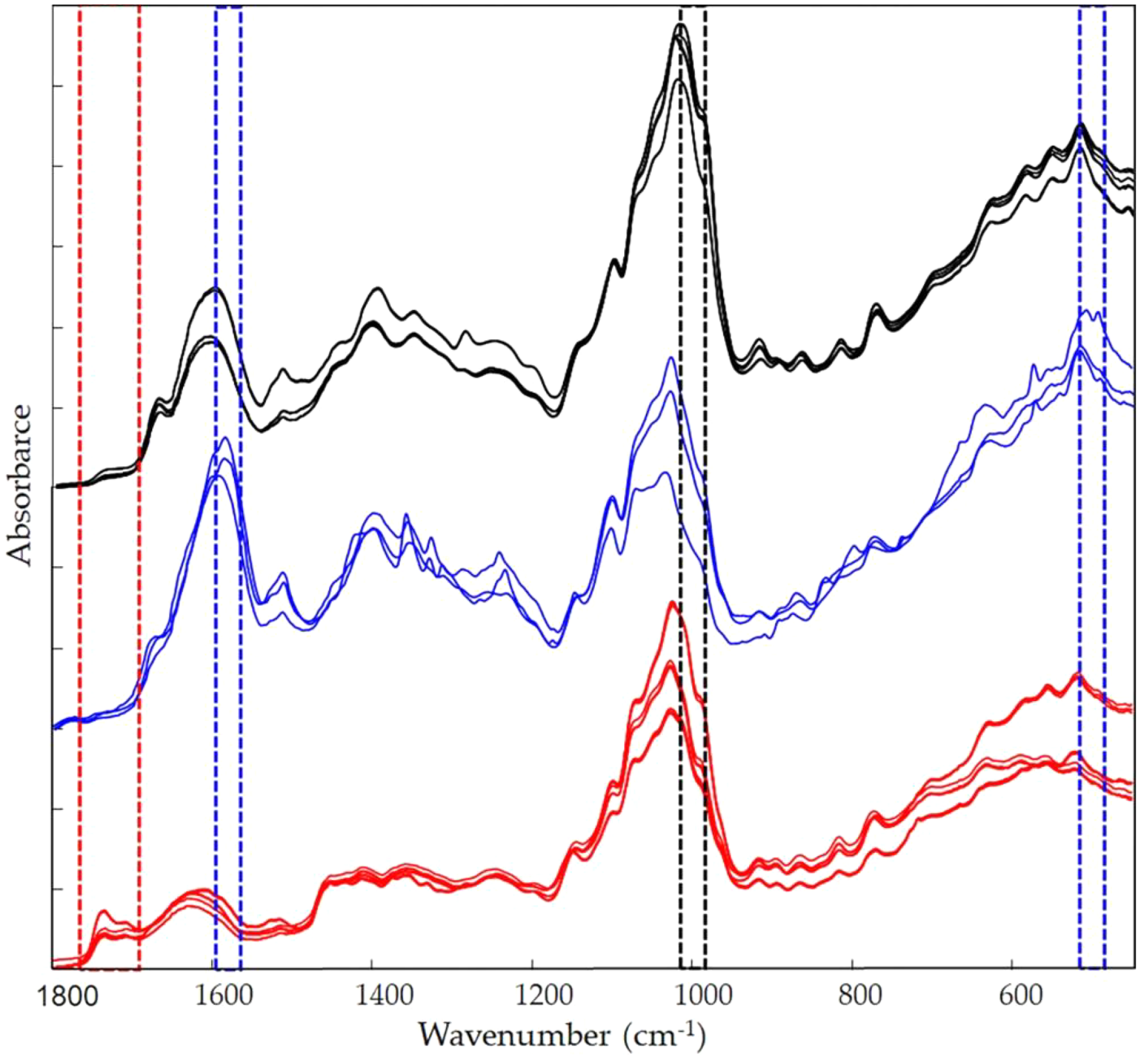
Infrared fingerprint characterization. FTIR spectra for the different extraction moieties: acetone extract (red lines); EtOH 70% extract (blue lines); MeOH extract (black lines).

**Figure 10:**
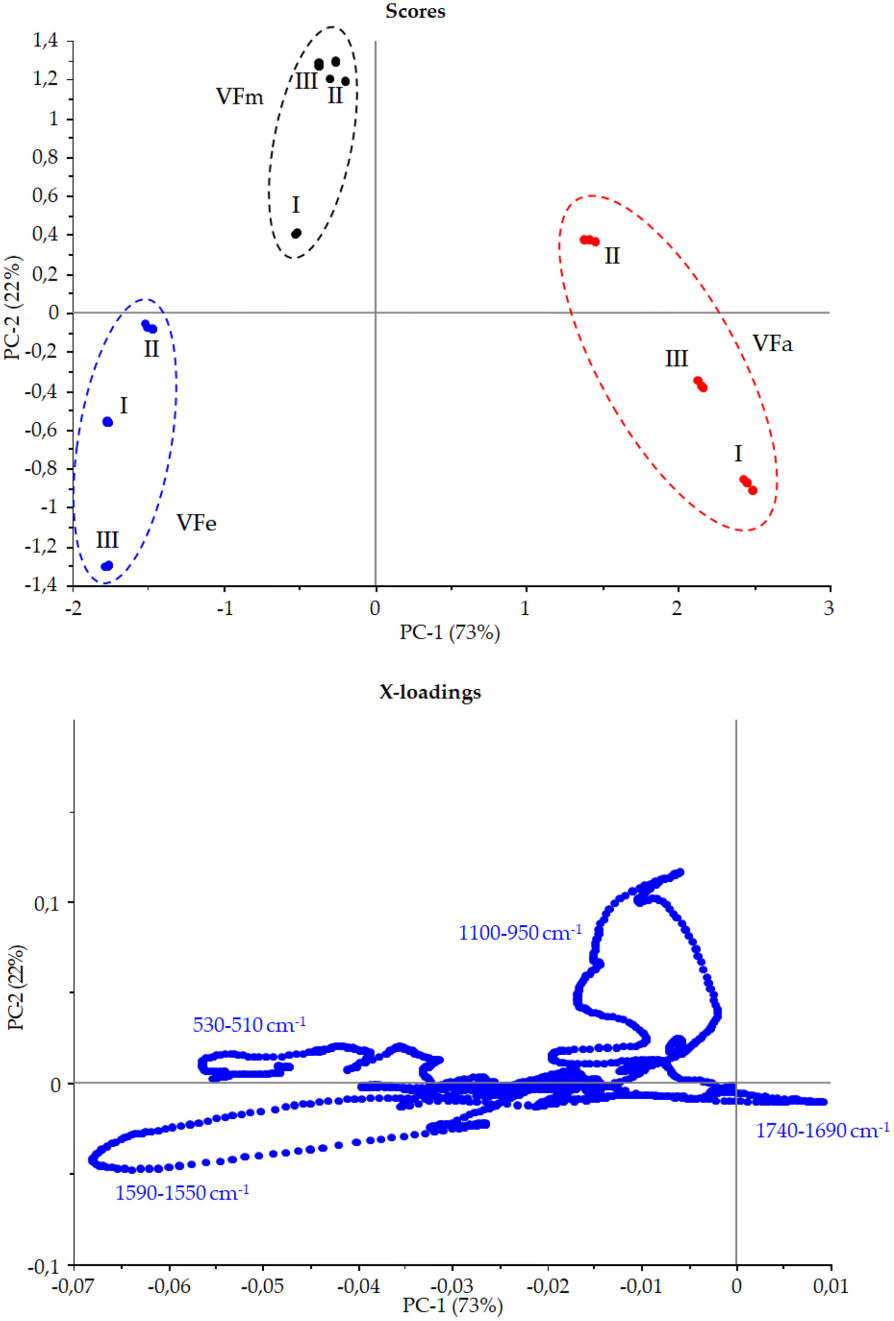
Principal component assay. Score and X-loadings plots, PC1 *vs* PC2 obtained in PCA modelling to FTIR data from VF extracts in the range 1,800–450 cm^−1^

Figure 9 shows the *Vicia faba* L. extracts spectra recorded from extraction procedures, where the common spectral bands associated with aromatic rings and phenol moieties are visible. The methoxy group present in some phenolic compounds showed signals between 1,450 and 950 cm^−1^. The phenolic acids were characterized by bands of unsaturated carboxylic acids (range 1,775–1,630 cm^−1^), while flavonoids moiety mainly by bands associated with benzopyrylium and benzo-γ-pyrone vibrations in the regions 1,650–1,400 and 1,200–450 cm^−1^. Phenolic compounds have a large and overlapped infrared fingerprint. The use of single peaks or limited wavenumber ranges to distinguish the different extraction procedures or ripening times seemed very difficult, making it necessary to use chemometric methodologies (Abbas et al., 2017). PCA elaboration was applied to the spectral data matrix, with the aim to discriminate the different metabolic contribution of *Vicia faba* L. bean samples. Figure 10 describes the plot scores by considering the first and second principal components with a total % explained variance of 95%. The sample grouping was evident and it was possible to distinguish the samples according to the extraction procedure and in some cases also the harvest time of the VFs. By comparing the X-loading values calculated for the elaborated waves with respect to the first two principal components (PCs), some characteristic peaks for each extraction technique were highlighted (Fig. 10). The correspondence between PCA and spectral fingerprint is shown in Fig. 9. PCA showed a relative abundance of information for the acetone extract in the spectral range 1,740–1,690 cm^−1^, and these characteristic bands can be explained when hydroxybenzoic and hydroxycinnamic acids are present in the samples. The MeOH extract was characterized by the bands in the range 1,100–950 cm^−1^, and the EtOH 70% extract in the ranges 1,590–1,550 and 530–510 cm^−1^, confirming the differences in phenols composition according to the chromatographic study.

### Effects on cancer cells viability of V.faba L. pods extracts

The cytotoxic effects of the *V.faba* L. pods extracts, at three different harvest periods, were examined against two human melanoma cell lines, A2058 and Sk-Mel-28, two human breast carcinoma cells, MCF-7 and MDA-MB-231, two uterine carcinoma cell lines, HeLa and Ishikawa, and the human colon carcinoma RKO cells using MTT assay. As normal cell lines, we adopted the human mammary epithelial cells, MCF-10A, and the human embryonic kidney epithelial cells, Hek-293. The IC_50_ values were determined after 72 h of treatment at different concentrations (50, 100, 200, 400 and 500 μg mL^−1^) of the acetone, methanol and ethanol VFs extracts and are reported in Table 4, together with those from Vinblastine, a well-known Vinca alkaloid used as reference molecule. The acetone extracts (VFIa, VFIIa and VFIIIa) resulted cytotoxic for all the cell lines used in these experiments, with few exceptions (Table 4). However, the lack of selectivity between cancer and normal cells make these extracts useless for further investigations. The three methanol and ethanol extracts exhibited, instead, a better cytotoxic profile and a selectivity between the cancer and normal cells. The VFIm extract was found to be particularly active toward the Sk-Mel-28 cells viability (IC_50_ of 120.8 ± 1.2 μg mL^−1^), with a lesser cytotoxicity against MCF-7 (IC_50_ of 392.8 ± 0.6 μg mL^−1^) and MDA-MB-231 (IC_50_ of 467.3 ± 0.9 μg mL^−1^) cells and lacked any effects on the other cell lines used, included the normal cells, MCF-10a and Hek-293. The VFIIm extract maintained a good anticancer activity against the Sk-Mel-28 cells, with an IC_50_ value of 170.9 ± 1.0 μg mL^−1^, but lost the activity against the breast cancer cells and resulted inactive toward all the other cells, included the normal ones. Finally, the VFIIIm extract exhibited a lesser activity against the Sk-Mel-28 cells (IC_50_ of 256.1 ± 0.8 μg mL^−1^) with respect to VFIIm and VFIm and gained a discrete activity against the MCF-7 cells (IC_50_ of 330.8 ± 1.3 μg mL^−1^); no cytotoxic activity was recorded against the other cell lines. The VFIe extract had the best activity against the Sk-Mel-28 cells, with an IC_50_ value of 118.7 ± 0.5 μg mL^−1^, a very low activity against the breast cancer cells (444.6 ± 1.2 and 403.0 ± 0.7 μg mL^−1^ for MCF-7 and MDA-MB-231 cells, respectively) and a discrete activity against the HeLa cells, being the only one active against this cancer cells line and showing no effects on the normal cells viability and on the other cancer cells lines used. The VFIIe showed an IC_50_ value of 175.1 ± 0.7 μg mL^−1^ against the Sk-Mel-28 cells, with a cytotoxic profile comparable to that of the VFIIm extract. Finally, the VFIIIe extract was found active only against the Sk-Mel-28 and MCF-7 cells, with IC_50_ values of 287.9 ± 1.3 and 326.7 ± 0.9 μg mL^−1^, respectively, similarly to VFIIIm. Summing up, the VF acetone extracts are not selective between cancer and normal cells, whereas the VF methanol and ethanol extracts affect particularly the Sk-Mel-28 cells viability, with very close IC_50_ values and following this trend in terms of activity: VFIe > VFIm > VFIIm > VFIIe > VFIIIm > VFIIIe. When compared to our extracts, Vinblastine exerted a stronger antitumor activity against all the used cancer cell lines, but accompanied by a dramatic cytotoxicity towards the normal cell lines (IC_50_= 17.2 × 10^−3^ ± 0.6 and 33.5 × 10^−3^ ± 1.0 μg mL^−1^ on MCF-10A and Hek-293, respectively). The VFIe extract is, overall, the most active of the series and is the only one that exhibited a discrete activity as well against the HeLa cells. Moreover, both the ethanol and methanol extracts did not affect the viability of the MCF-10A and Hek-293 normal cells.

**Table 4:** IC_50_ values of VF extracts. IC_50_ values of VF extracts in three different solvents (a = acetone, m = MeOH, e = EtOH) at three different collection times (I, II and III).

IC_50_ (μg mL^−1^)									
	**A2058**	**Sk-Mel28**	**MCF-7**	**MDA-MB-231**	**HeLa**	**Ishikawa**	**RKO**	**MCF-10A**	**Hek-293**
**VFIa**	126.0 ± 0.4	172.5 ± 1.1	278.3 ± 0.8	270.8 ± 1.3	253.6 ± 0.8	>500	>500	87.3 ± 0.7	24.4 ± 0.9
**VFIIa**	218.5 ± 0.9	383.6 ± 0.9	>500	>500	>500	>500	>500	340.4 ± 1.1	497.4 ± 1.0
**VFIIIa**	121.0 ± 1.1	328.3 ± 1.2	318.5 ± 0.9	229.5±1.0	132.6 ± 0.6	>500	>500	82.2 ± 0.6	253.8 ± 1.2
**VFIm**	>500	120.8 ± 1.2	392.8 ± 0.6	467.3±0.9	>500	>500	>500	>500	>500
**VFIIm**	>500	170.9 ± 1.0	>500	>500	>500	>500	>500	>500	>500
**VFIIIm**	>500	256.1 ± 0.8	330.8 ± 1.3	>500	>500	>500	>500	>500	>500
**VFIe**	>500	118.7 ± 0.5	444.6 ± 1.2	403.0±0.7	209.0 ± 0.8	>500	>500	>500	>500
**VFIIe**	>500	175.1 ± 0.7	>500	>500	>500	>500	>500	>500	>500
**VFIIIe**	>500	287.9 ± 1.3	326.7 ± 0.9	>500	>500	>500	>500	>500	>500
**Vinblastine**	1.6 × 10^−3^ ± 1.2	1.9 × 10^−3^ ± 0.9	36.8 × 10^−3^ ± 0.7	130.2 × 10^−3^ ± 1.1	55.3 × 10^−3^ ± 0.9	21.4 × 10^−3^ ± 0.7	629.7 × 10^−3^ ± 1.3	17.2 × 10^−3^ ± 0.6	33.5 × 10^−3^ ± 1.0

### Cells morphology changes and death by apoptosis

**Figure 11:**
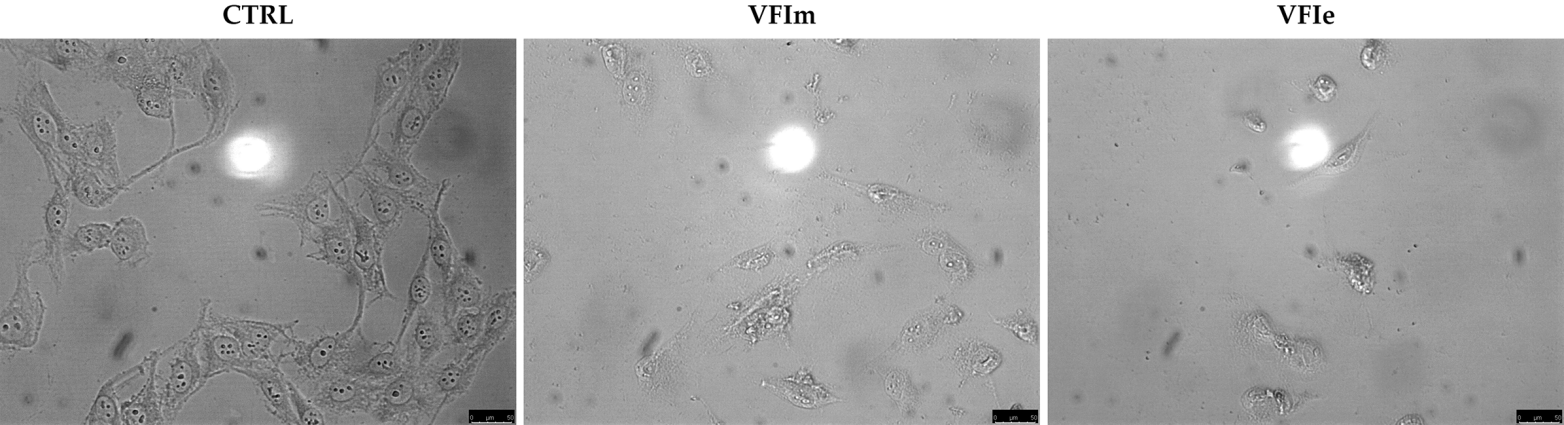
Cells morphology changes. Exposure of Sk-Mel-28 cells to the methanol and ethanol 70% extracts from VFI (VFIm and VFIe), induced dramatic changes in cell morphology: cells become round and shrunk already after 24 h. The cells were imaged under an inverted microscope (20× magnification).

Sk-Mel-28 cancer cells were used for the next investigations, being particularly sensitive to the VFIe and VFIm. During the viability assays, we noticed a dramatic cell morphology change and an elevated number of floating cells, suggesting that they were dying (Fig. 11, VFIm and VFIe), whereas the vehicle-treated cells were adherent and possessed a normal polygonal morphology (Fig. 11, CTRL).

**Figure 12:**
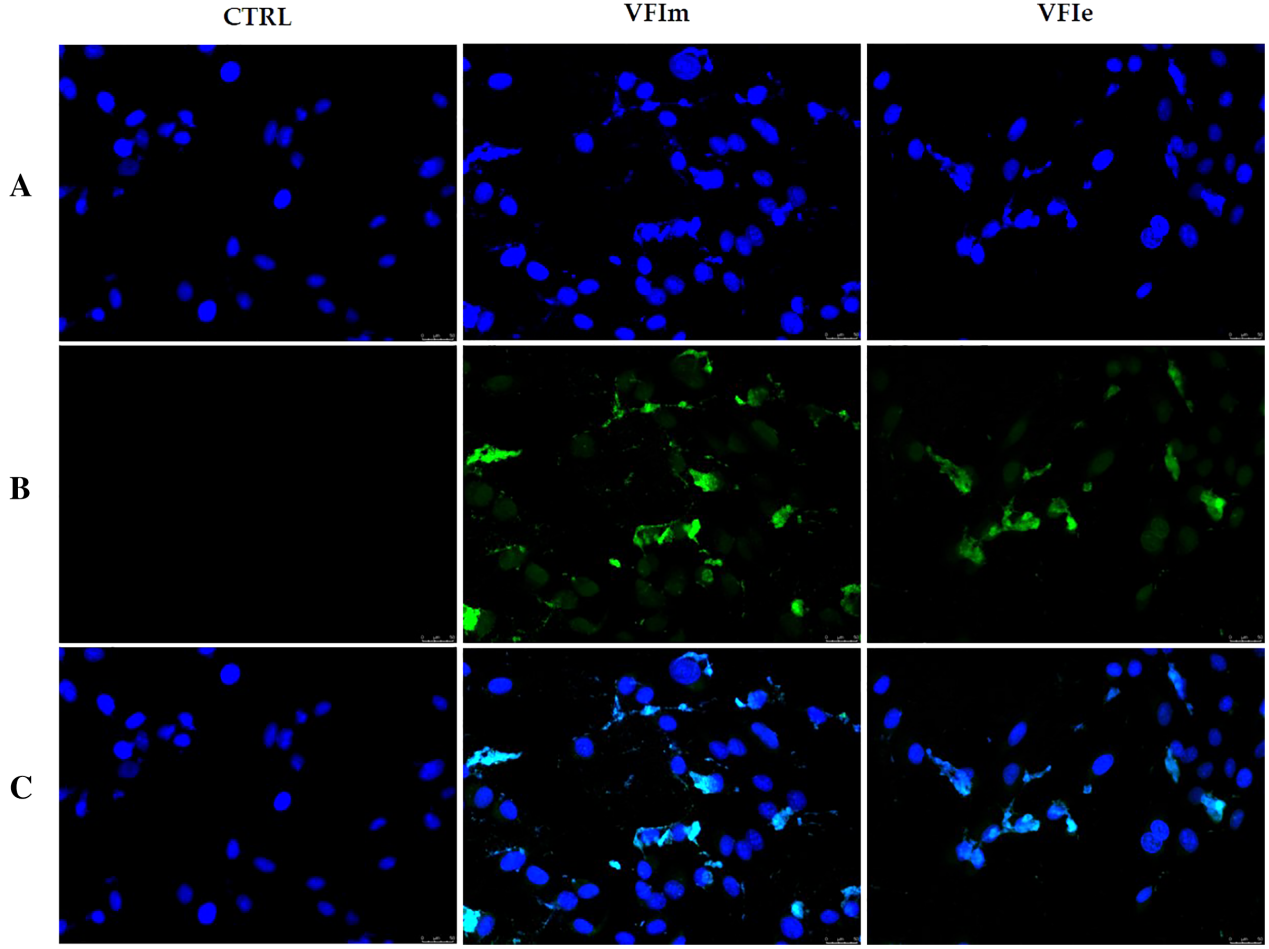
TUNEL assay. Sk-Mel-28 cells have been treated with *V. faba* L. broad bean pods methanol and ethanol 70% extracts at their first harvest period (VFIm and VFIe, at their IC_50_ values) for 24 h or vehicle (CTRL). After treatment, cells have been cold methanol fixed and subjected to TdT reaction (see the experimental section for more details). Then, cells have been dyed with DAPI and observed and imaged under an inverted fluorescence microscope (20× magnification). (A) CF™488A λ_ex/em_ = 490/515 nm; (B) DAPI and λ_ex/em_ = 350/460 nm; (C) overlay. Representative fields have been shown.

In order to characterize the cell death at the molecular level, we performed a TUNEL assay after 24 h of cells exposure to VFIe and VFIm extracts, used at their IC_50_ values. Figure 12 shows that in the VFIe and VFIm extracts-treated cells is visible a green fluorescence localized in the cell nuclei (VFIm and VFIe, Fig. 12B) and not detectable in the vehicle-treated cells (Fig. 12B, CTRL), indicating DNA fragmentation and apoptosis.

### Effects of methanol and ethanol V.faba L. pods extracts on cell microtubules

**Figure 13:**
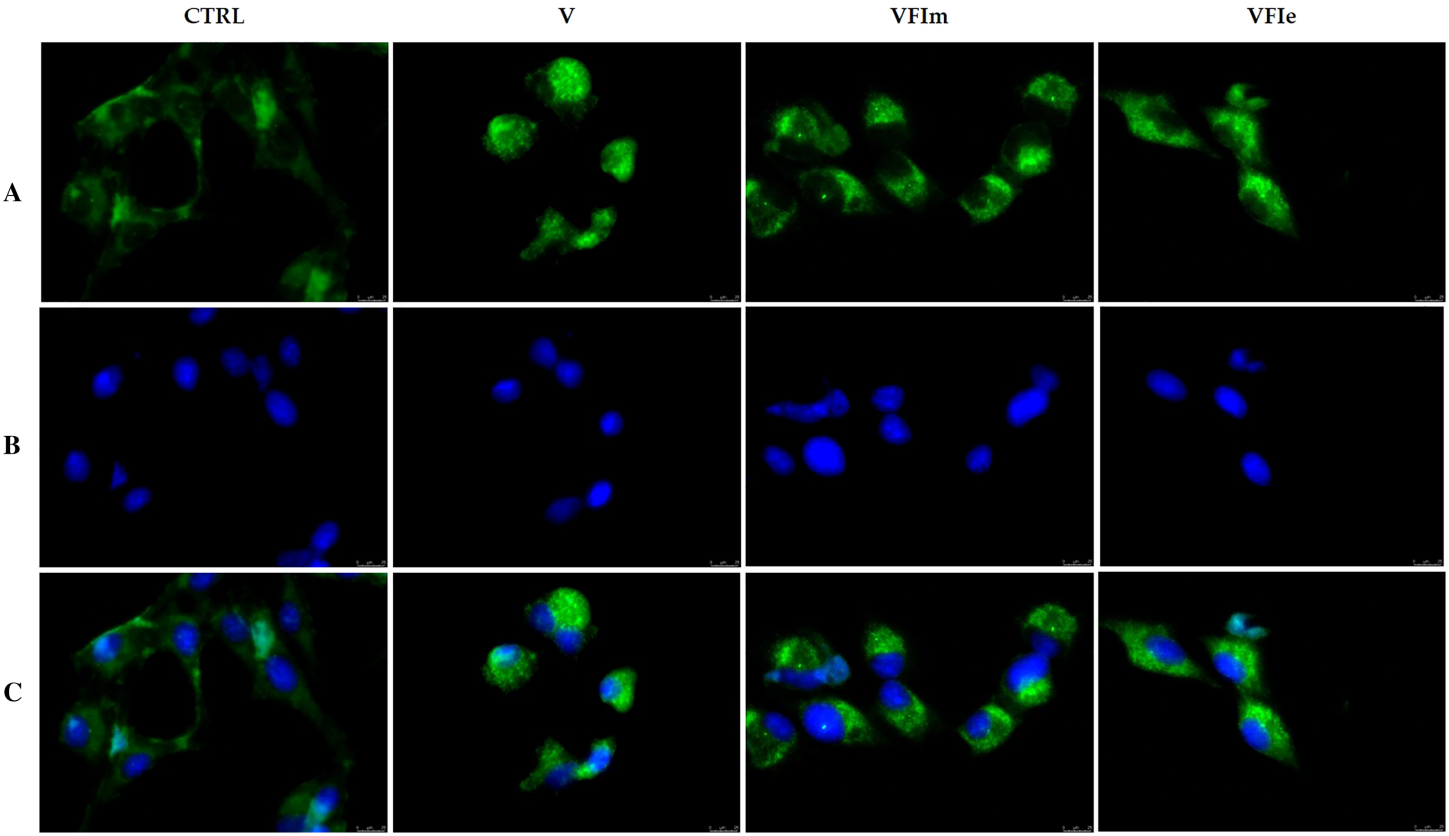
Immunofluorescence studies. Sk-Mel-28 cells were treated with Vinblastine (used as reference molecule) at its IC_50_ value, with VFIm and VFIe extracts or with a vehicle (CTRL) for 24 h. After treatment, the cells were incubated with primary and secondary antibodies (see Experimental section for more details) and then imaged under the inverted fluorescence microscope at 40x magnification. CTRL cells showed a regular tubulin organization (A, CTRL). Microtubules of Sk-Mel-28 cells treated with vinblastine (A, V) and methanolic and ethanolic extracts from VFI (A, VFIm and VFIe) resulted packed and dotted near the nuclei. (A) tubulin (Alexa Fluor® 568) λ_ex/em_ = 644 nm/665 nm; (B) DAPI, λ_ex/em_ = 350 nm/460 nm; (C) a merge. Representative fields have been shown.

The morphological cells changes observed and the detected cell death by apoptosis pushed us to further investigate the mechanism(s) by which our extracts could triggers these effects. Considered the earlier observation, we carried out immunofluorescence assays on tubulin of the Sk-Mel-28 cells exposed to VFIe and VFIm extracts or only vehicle (negative control) for 24 h. As positive control we adopted one of the most know drugs targeting the tubulin, *i.e*. the vinblastine, able to inhibit the polymerization reaction. The obtained results are provided in Fig. 13, where the vehicle treated cells showed a normal tubulin organization, the microtubules are fairly distributed throughout the cell cytoplasm and sharply defined (CTRL, panel A). Conversely, under the vinblastine treatment (Fig. 13B, V) the microtubules organization seemed completely altered: indeed, there is a brighter fluorescence unevenly gathered around the cells nuclei, with a dot-like aspect due to the crystals and para-crystals formation. As well for the VFIe and VFIm treated cells, a similar result has been obtained and the microtubules resulted packed and dotted near the nuclei (Fig. 13A, VFIm and VFIe). Finally, we can deduce that one of the intracellular targets of the two above-mentioned extracts are the microtubules, which structural alteration results in a dysfunctional cell cytoskeleton responsible of the observed apoptotic death.

### Docking simulations

We used molecular docking simulations to evaluate the possible binding modes of our phenolic compounds identified in *Vicia faba* L. extracts by HPLC and to calculate their binding affinities to a tetrameric assembly of tubulin. We initially performed a blind-docking using the program Vina (Trott & Olson, 2010) in order to identify the binding sites and then, through the program Autodock 4.2.2 (Morris et al., 2009) we calculated the affinities of the compounds to the protein target, in our case we used the three-dimensional structure of tubulin in complex with Vinblastine (Waight et al., 2016). The protocol we adopted for the simulations is the one we discussed in several other previous works (Abbassi et al., 2014; Rosano et al., 2016; Sinicropi et al., 2018) that allows us to identify the best binding mode basing on the clusterization of the results of our simulations together with the visual inspection of the binding area.

**Figure 14:**
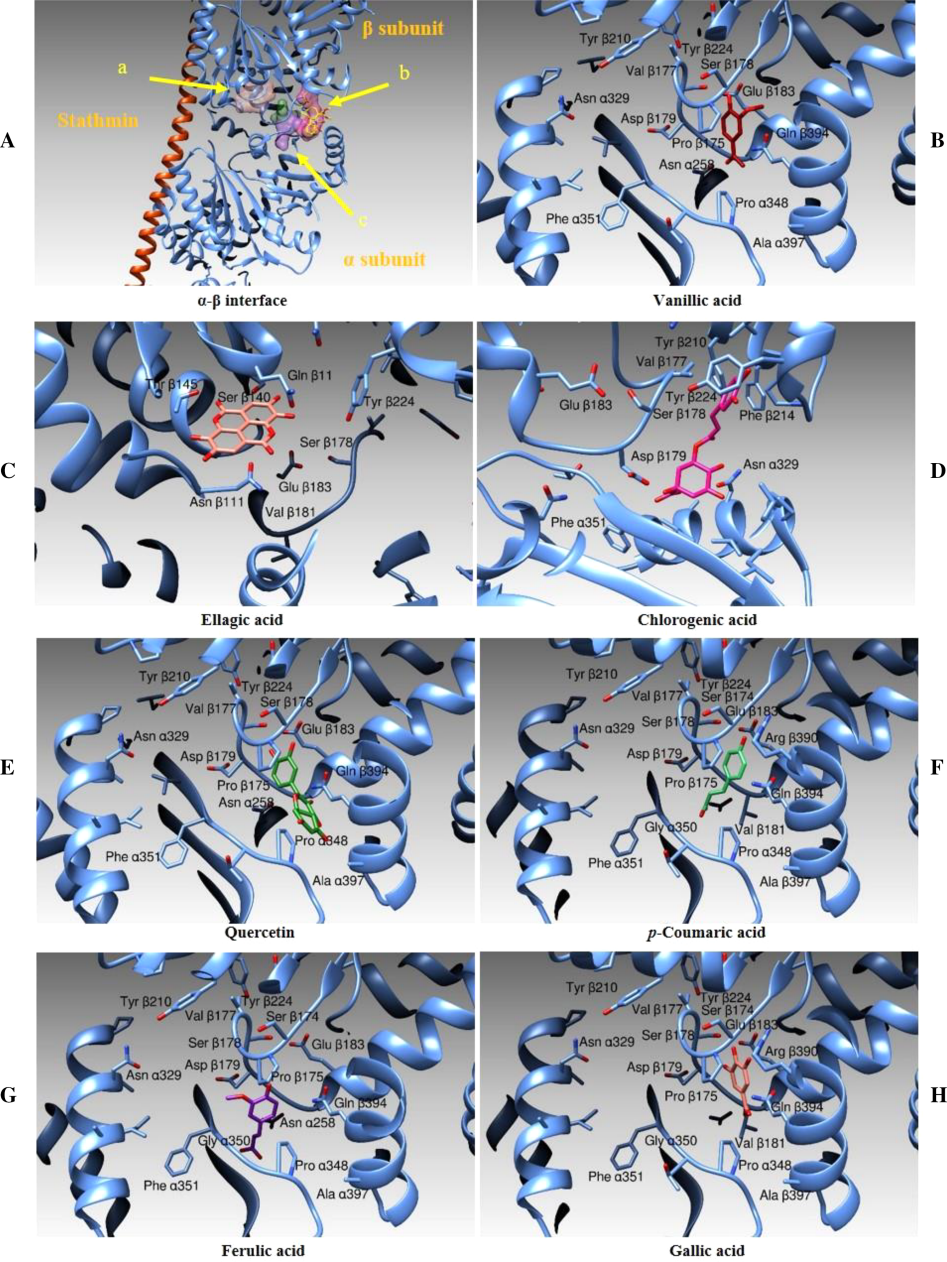
Docking Simulations. Representation of the different binding modes of the phenolic compounds from *Vicia Faba* L. extracts. (A) The interface between two monomers in the tetrameric assembly of tubulin, as determined by X-ray crystallography. The stathmin protein is drawn as an orange ribbon, the α and β-tubulin subunits are illustrated as cyan ribbons. The binding areas for ellagic acid (A), chlorogenic acid (B) and the other tested compounds (C) are evidenced by colored surfaces (orange, purple and green-violet, respectively). (B–H) The different compounds in their binding modes as determined by docking simulations.

In this case, we identified three main binding zones, as shown in Fig. 14A. A first binding site (shown as an orange transparent surface) is occupied by ellagic acid and it is superposed to the binding site of a GDP molecule in the three-dimensional structure of tubulin in complex with vinblastine. A second area (purple transparent surface) is correspondent to the vinblastine binding cleft and, in our simulations, is occupied by the chlorogenic acid moiety. A third binding zone (green-violet surface) is the binding zone of the other compounds we tested (quercetin, and vanillic, gallic, ferulic and *p*-Coumaric Acids). Figure 14B–14H and Table 5 show the interactions between all the above-mentioned ligands and the protein amino acids in deeper details. Table 5 also reports the binding energies and the calculated affinity constant for each compound.

**Table 5:** List of ligand:protein interactions. Interactions with the protein amino acids, calculated affinity constant and binding energies for all the compounds tested *in silico* towards tubulin.

Compounds	Polar interactions	Hydrophobic interactions	Ki (10^−6^ M)*	Binding Energies (Kcal/mole)
**Chlorogenic Acid**	Asp α329 Phe α351 N-pept. Asp β179 Phe β214 COO^-^ Tyr β224	Val β177 Phe β214	21.6	−6.36
**Ellagic Acid**	Gln β11 Glu β71 Ser β140 Thr β145		0.914	−8.24
**Ferulic Acid**	Gly α350 COO^-^	Pro α348 Pro β175 Val β181	20.95	−6.38
**Gallic Acid**	Ser β178 Glu β183 Arg β390 Gln β394	Pro α348 Pro β175 Val β181	30.69	−6.16
**p-Cumaric Acid**	Asn α258 Gly α350 COO^-^ Ser β174 N-pept. Arg β390	Pro α348 Pro β175 Val β181	30.37	−6.16
**Quercetin**	Ser β174 N-pept. Ser β178 Glu β183 Glu β393 Gln β394	Pro α348 Ala α397 Pro β175 Val β181	0.523	−8.57
**Vanillic Acid**	Glu β183 Gln β394	Pro α348 Pro β175 Val β181	104.87	−5.43

**Note:**

* Ki = exp (deltaG/(R × T)).

Taking into consideration the analysis of the clusterization of the results and the visual inspection of the molecular binding modes, the best among the ligand tested seems to be quercetin. Its binding to the beta subunit should prevent the stacking of a subsequent alpha subunit inhibiting therefore the correct polymerization of the assembly. Vanillic and gallic acids should act the same way of quercetin although with less efficiency being these ligands shorter. Ellagic Acid should interfere with the correct functioning of the tubulin assembly being its binding site superposed to the to the site dedicated to bind the GDP moiety. Ferulic, chlorogenic and *p*-Coumaric acids are positioned between the two tubulin monomers and they act as a link between the subunits α and β and thus they should prevent the correct depolymerization of the microtubular assembly.

## Discussion

Very often, the peels of vegetables or fruits are discarded or, at most, employed as fertilizers or for cattle feeding, creating significant issues for the environment (L’Hocine et al., 2020). More recently, a turnabout promoted by several scientific studies, directed towards the re-evaluation and the valorization of agro-food wastes, has been recorded. Indeed, many literature data reported the high content in bioactive compounds of some by-products, very important as nutraceuticals with countless benefits for human health, included the antioxidant and anticancer ones (Batra & Sharma, 2013). Herein, we proposed a valorization of the *Vicia faba* L. pods as source of antioxidant and anticancer compounds, investigating these properties at three different times of collection, with an interval of 20 days from each other, and using three solvents (acetone, MeOH and EtOH 70%. The selected solvents aimed at a preliminary efficient recovery of glucans (Fazio et al., 2020). The water content relative to pods from the three collection times increased with the ripening and is shown in Fig. 1, whereas the quantitative recovery for each stage and solvent used for the extraction is reported in Fig. 2. The higher amounts were obtained in the methanol extraction (particularly from VFII), whereas the acetone one produced the lowest recovery. The resulted TPC content, shown in Fig. 3, followed this trend: VFIIa > VFIe > VFIm > VFIIe > VFIIIe > VFIa > VFIIIm > VFIIm > VFIIIa, thus the increasing solvent polarity is related to the phenolic content increase, with the only exception of the second collection stage, where the acetone extract has the highest content. Conversely, the TFC content exhibited an overall inverse relation with respect to the extraction solvent polarity and ripening progression, as follows: VFIa > VFIIa > VFIIIa > VFIIm ≈ VFIm > VFIIIm = VFIIIe > VFIIe ≈ VFIe, being acetone the solvent with the higher extraction efficacy (Fig. 4). Several literature data reported that legumes are an excellent source of nutrients, included secondary metabolites such as polyphenols, whose biological properties have been widely studied (Perez-Jimenez et al., 2010). In particular, these molecules possesses well-recognized antioxidants properties thanks to their ability to neutralize the reactive oxygen/nitrogen species produced as byproduct during metabolic processes (Wink, 2015). In addition, several studies have demonstrated that polyphenols provide a significant protection against chronic diseases such as cardiovascular diseases, diabetes, infections, aging, asthma and also cancer (Pandey & Rizvi, 2009). Different factors influence the amount of these phytochemicals, as the genotype and the growing stage, thus we investigated the antioxidant ability of our extracts using the DPPH and ABTS assays. As expected, the first stage of collection exhibited the highest DPPH scavenger ability, being the methanol the best antioxidant extract. As well, the ABTS assay indicated the aqueous ethanol extract from VFI as the most effective in radical scavenging. Overall, these results agreed that VFIm and VFIe were the most efficient extracts in inhibiting DPPH and ABTS radical by 50% for the numerous hydroxyl groups in the structure of the present phenolic compounds. In order to go in deep with regard to the phenolic and flavonoid content, we identified and quantified seven compounds, by HPLC-DAD analyses of all the extracts. As visible in Fig. 8, the heat map showed that the *p*-coumaric acid is the more represented in all the extracts, with some differences related to the collection time, whereas the ellagic acid is the less represented. However, accordingly with our previous observations, the VFI extracts exhibited, quantitatively and qualitatively, the best polyphenolic profile. A recent trend exploiting some spectroscopic techniques, able to detect and characterize the phenolic and flavonoid content profile in different vegetal samples has been recorded. The versatile FT-IR techniques, for instance, can detect the chemical structure of phenolic compounds because of their vibrational molecular motions in specific ranges and, together with the evaluation of PCA, represent a successful and more rapid detection method (Holser, 2012). Following this trend, FT-IR spectra have been recorded from the *Vicia faba* L., extracted with the adopted solvents at the above-mentioned collection times, and reported in Fig. 9. PCA elaboration was applied to the spectral data matrix and the plot scores, depicted in Fig. 10, indicating distinct samples grouping that agree with the extraction procedures and the ripeness of the VFs samples. Additionally, the data from PCA and spectral fingerprint revealed the presence of hydroxybenzoic and hydroxycinnamic acids in the acetone extracts supporting the HPLC-DAD results. Since polyphenols are also associated with interesting anticancer properties, we next focused our attention on the evaluation of the anticancer activity of our extracts on a panel of different cancer cell lines and on the cytotoxic effect against the growth the MCF-10A and Hek-293 normal cell lines. The extracts exhibited strong anticancer properties on the adopted cancer cells, with some exceptions. The acetone extracts from all the three maturation stages exhibited a non-selective cytotoxicity, therefore they were not considered for further investigations. Conversely, all the methanol and aqueous ethanol extracts were selective between cancer and normal cells and particularly active against the melanoma Sk-Mel-28 cells, being the VFIe and VFIm the most active of the series, without affecting the growth of the two normal cell lines used. The anticancer activity of these extracts could be due to their best qualitative and mostly quantitative polyphenolic profile. Indeed, the anticancer activity against different cell lines of *p*-coumaric and ferulic acids, most present in VFIe and VFIm extracts, is well-known (Anantharaju et al., 2016). As reference molecule we used vinblastine, another plant derived anticancer compound, which showed a higher anticancer activity but, as well, a dramatic impact on the growth of the normal cells, contrarily to VFIe and VFIm. The clear change in cancer cells morphology under the exposure to VFIe and VFIm, induced us to suppose the apoptotic death, as demonstrated by TUNEL assay (Fig. 12B). Changes in cell morphology can be ascribed to various factors, amongst them a possible interference of the extracts with the cell cytoskeleton. Thus, we performed immunofluorescence assays on the Sk-Mel-28 cells in order to visualize the cell microtubules, under the two extracts exposure and, as positive control, vinblastine, known to inhibit the polymerization reaction. In the DMSO-treated cells (Fig. 13A, CTRL) the dendritic structures are preserved. Under VFIm and VFIe treatment (Fig. 13A, VFIm and VFIe), a brighter fluorescence and the presence of dotted crystals and para-crystals around the cells nuclei suggested a loss of the microtubules organization. Similar results were obtained under vinblastine exposure (Fig. 13A, V). It should be emphasized that, under VFIe and VFIm extracts or vinblastine treatment, the microtubules within the Sk-Mel-28 cells dendritic structures disappeared, and that microtubules, amongst the vital cell functions, are involved in the intracellular transport of melanosomes and essential for both their dispersion or aggregation (Logan, Burn & Jackson, 2006; Wu & Hammer, 2000). *In. silico* studies demonstrated that all the phenolic compounds identified in *Vicia faba* L. extracts by HPLC were able to bind with good binding energies the three-dimensional structure of tubulin. The high presence in *p*-coumaric, ferulic acids in both the VFIm and VFIe extracts could be linked to the observed ability of these extracts to interfere with the tubulin. They are positioned between the two tubulin monomers and could prevent the correct depolymerization of the microtubular assembly, as supposed by docking studies. Summing up, the two extracts from *Vicia faba* L. were proved to possess antioxidant and anticancer properties and that the observed effect against the melanoma Sk-Mel-28 cells was due to the alteration of tubulin cytoskeleton organization, leading to apoptosis.

## Conclusions

The sustainable and effective valorization of agro-food of wastes/by-products to obtain value-added products provides an opportunity for pharmaceutical applications and can reduce the environmental stress by decreasing unwanted pollution. With this in mind, different *Vicia faba* L. pods extracts obtained using three different solvents (acetone, MeOH and EtOH 70%: VFa, VFm, and VFe, respectively), at three different collection times (VFI, VFII and VFIII), were studied. These extracts showed an interesting antioxidant ability, studied *via* DPPH and ABTS assays, higher for the first stage of collection, especially for VFe. Regarding the anticancer activity, VFIe and VFIm were particularly active against the melanoma Sk-Mel-28 cells and not cytotoxic against normal cell lines. Immunofluorescence and *in silico* studies showed that the observed effect against the melanoma cells was mainly due to the alteration of tubulin cytoskeleton organization, triggering apoptosis. The antioxidant and anticancer activity of VFIe and VFIm could be related to their best qualitative and mostly quantitative polyphenolic profile. Thus, this article gives some hints for the valorization of *Vicia faba* L. pods as sources of antioxidant and anticancer molecules, enlarging the knowledge about the properties of this legume.

## Supplemental Information

10.7717/peerj.13683/supp-1 Supplemental Information 1 *Vicia faba* L. pods extracts yields.

10.7717/peerj.13683/supp-2 Supplemental Information 2 Raw data: determination of IC50.

10.7717/peerj.13683/supp-3 Supplemental Information 3 Raw data: water content and calibration curves.

## Abbreviations

EtOH: 70% (ethanol:water 70:30)
MeOH: (methanol)
DMSO: (dimethylsulfoxide)
GA: (gallic acid); QE (Quercetin)
TPC: (Total phenolic content)
TFC: (Total flavonoid content)
TE: (Trolox equivalent)
DPPH: (2,2′-diphenyl-1-picrylhydrazyl)
ABTS: (2,2′-azino-bis (3-ethylbenzothiazolin-6-sulfonic))
Trolox: (6-hydroxy-2,5,7,8-tetramethylcroman-2-carboxylic acid)
HPLC: (High Performance Liquid Chromatography)
VF: (*Vicia faba* L. pods)
VFI: (*Vicia faba* L. pods of first collection)
VFII: (*Vicia faba* L. pods of second collection)
VFIII: (*Vicia faba* L. pods of third collection)
VFIa, VFIIa, VFIIIa: (acetone extracts from VFI, VFII and VFIII, respectively)
VFIm, VFIIm, VFIIIm: (methanolic extracts from VFI, VFII and VFIII, respectively).
VFIe, VFIIe, VFIIIe: (aqueous ethanolic extracts from VFI, VFII and VFIII, respectively)
FBS: (Fetal Bovine Serum)
HS: (Horse Serum)
MTT: (3-(4,5-dimethylthiazol-2-yl)-2,5-diphenyl tetrazolium bromide)
DAPI: (4’,6-diamidino-2-phenylindole)
TUNEL: (terminal deoxynucleotidyl transferase dUTP nick end labeling)
DMSO: (Dimethyl sulfoxide)

## Additional Information and Declarations

### Data Availability

The following information was supplied regarding data availability: The raw data is available in the Supplemental Files.
