# Supplementary material for: Exploring the anticancer and antioxidant properties of Vicia faba L. pods extracts, a promising source of nutraceuticals
Source: PeerJ. 2022 Aug 17;10:e13683. doi: 10.7717/peerj.13683 (PMC9392456; doi:10.7717/peerj.13683)
Supplement: Supplemental Information 1 [file peerj-10-13683-s001.docx]

| **Samples: 3g** | **VFI** | **VFII** | **VFIII** |
| --- | --- | --- | --- |
| **Acetone*** | 67.2 ± 0.7 mg  (yield: 2,24%) | 99 ± 1.2 mg  (yield: 3.3 %) | 68.4 ± 1.1 mg  (yield: 2.28%) |
| **MeOH*** | 710.4 ± 3.7 mg  (yield: 23,68%) | 954 ± 2.3 mg  (yield: 31.8%) | 889.2 ± 2.8 mg  (yield: 29.64%) |
| **EtOH 70%*** | 194.4 ± 1.8 mg  (yield: 6.48%) | 231 ± 3.1 mg  (yield: 7.7%) | 326.4 ± 1.5 mg  (yield: 10.88%) |

***Vicia faba* L. pods extracts yields**

* 30 mL/3g
